# The evolutionary history of three *Baracoffea* species from western Madagascar revealed by chloroplast and nuclear genomes

**DOI:** 10.1371/journal.pone.0296362

**Published:** 2024-01-11

**Authors:** Rickarlos Bezandry, Mathilde Dupeyron, Laura Natalia Gonzalez-Garcia, Artemis Anest, Perla Hamon, Hery Lisy Tiana Ranarijaona, Marie Elodie Vavitsara, Sylvie Sabatier, Romain Guyot

**Affiliations:** 1 École Doctorale sur les Écosystèmes Naturels (EDEN), Mahajanga, Madagascar; 2 Faculté des Sciences de Technologie et de l’Environnement (FSTE), Université de Mahajanga, Mahajanga, Madagascar; 3 UMR DIADE, IRD, CIRAD, Université de Montpellier, Montpellier, France; 4 Systems and Computing Engineering Department, Universidad de los Andes, Bogotá, Colombia; 5 AMAP, CIRAD, CNRS, INRAE, IRD, Univ Montpellier, Montpellier, France; University of Veterinary Medicine Vienna: Veterinarmedizinische Universitat Wien, AUSTRIA

## Abstract

The wild species of the *Coffea* genus present a very wide morphological, genetic, and biochemical diversity. Wild species are recognized more resistant to diseases, pests, and environmental variations than the two species currently cultivated worldwide: *C*. *arabica* (Arabica) and *C*. *canephora* (Robusta). Consequently, wild species are now considered as a crucial resource for adapting cultivated coffee trees to climate change. Within the *Coffea* genus, 79 wild species are native to the Indian Ocean islands of Comoros, Mayotte, Mauritius, Réunion and Madagascar, out of a total of 141 taxa worldwide. Among them, a group of 9 species called "Baracoffea" are particularly atypical in their morphology and adaptation to the sandy soils of the dry deciduous forests of western Madagascar. Here, we have attempted to shed light on the evolutionary history of three Baracoffea species: *C*. *ambongensis*, *C*. *boinensis* and *C*. *bissetiae* by analyzing their chloroplast and nuclear genomes. We assembled the complete chloroplast genomes *de novo* and extracted 28,800 SNP (Single Nucleotide Polymorphism) markers from the nuclear genomes. These data were used for phylogenetic analysis of Baracoffea with *Coffea* species from Madagascar and Africa. Our new data support the monophyletic origin of Baracoffea within the *Coffea* of Madagascar, but also reveal a divergence with a sister clade of four species: *C*. *augagneurii*, *C*. *ratsimamangae*, *C*. *pervilleana* and *C*. *Mcphersonii* (also called *C*. *vohemarensis)*, belonging to the Subterminal botanical series and living in dry or humid forests of northern Madagascar. Based on a bioclimatic analysis, our work suggests that Baracoffea may have diverged from a group of Malagasy *Coffea* from northern Madagascar and adapted to the specific dry climate and low rainfall of western Madagascar. The genomic data generated in the course of this work will contribute to the understanding of the adaptation mechanisms of these particularly singular species.

## Introduction

By 2030, global temperatures are projected to increase by 1.5°C [1] in the best-case scenario. This temperature increase will lead to substantial global environmental alterations, thereby strongly impacting crop yields through more intense biotic and abiotic stresses [2]. Among crops of high economic significance, coffee tree (genus *Coffea*, family Rubiaceae) provides a livelihood for over 100 million people [3] and is an important part of the economy of many countries in southern regions. Currently, two *Coffea* species dominate the market: *C*. *canephora* Pierre ex A.Froehner (also called Robusta or Conilon), a diploid species cultivated since the 1850s [4] and accounting for around 40% of the market, and *C*. *arabica* L. (Arabica), an allotetraploid species cultivated for several hundred years and accounting for 60% of the production [5]. Both species seem particularly sensitive to climate change [6]. Specifically, *C*. *arabica*, native to Ethiopia, is a high-altitude plant adapted to temperatures of 18–23°C. It is acknowledged to be sensitive to climatic variations [7]. An illustrative case relates to Arabica yields (*C*. *arabica*) in Tanzania which experienced a decline of 137 kg per hectare for every degree increase in night-time temperature [8]. On the other hand, *C canephora* shown a greater tolerance compared to Arabica species, occupying ecological niches with temperatures ranging from 22°C to 30°C. However, it has been established that the optimal growth temperature for Robusta is only 20°C, and that temperatures variations of 1°C below or above a range of 16–24°C result in a 14% loss of production [9]. Considering the expected global changes, estimations indicate that the arable area under cultivation will shrink by almost 50% by 2050 [6]. In addition, four of the world’s top five coffee producers (Brazil, Vietnam, Colombia, and Indonesia) are threatened with a profound decline in the size and suitability of their best growing areas.

In the past, interspecific hybridization has been proposed and implemented within breeding programs (e.g., Arabusta, *C*. *arabica x C*. *canephora*; Congusta, C. *canephora x C*. *congensis*; and Aramosa, *C*. *arabica x C*. *racemosa*), to create coffee varieties with enhanced resistance to global change. Nowadays, the economic stakes and threats to Arabica and Robusta production have brought the historical works and practices on interspecific hybridization with wild species back to the fore. Fundamental knowledge of the wild species within the *Coffea* genus is an essential preliminary step before any attempt to use them in breeding. However, the potential of wild *Coffea* species to adapt to contrasted environmental factors remains poorly explored.

The *Coffea* genus currently comprises 130 recognized species, but this number rises up to 141 when all the taxa are included [10], most of which are cross-pollinated and distributed in Africa, Madagascar, the Comoros, the Mascarene Islands and Australasia [11, 12]. Some of these such as *C*. *liberica*, *C*. *dewevrei*, *C*. *stenophylla* [13], C. *mauritiana*, *C*. *congensis*, *C*. *racemosa*, *C*. *zanguebariae*, *C*. *bengalensis*, *C*. *travancorensis*, *C*. *wightiana* [14], *C*. *eugenioides* and *C*. *humblotiana* are or were consumed in the past, then abandoned for various reasons [15]. In addition to improving the quality of the beverage derived from the wild species seeds, they can also display exceptional environmental adaptation characteristics [16], such as tolerance to orange rust (Coffee Leaf Rust or CLR, *C*. *liberica*), drought tolerance (*C*. *stenophylla* [13] or *C*. *racemosa* [17]), highly variable fruit ripening times (from 4 to 14 months [18]) and agronomic and biochemical characteristics such as the absence of caffeine [15] in the seeds. However, there are major threats to these wild species and at least 60% of them are threatened with extinction due to anthropogenic activities resulting in a reduction of their habitat range. This includes species of potential interest for the improvement of cultivated coffee plants in the face of climate change.

**Fig 1:**
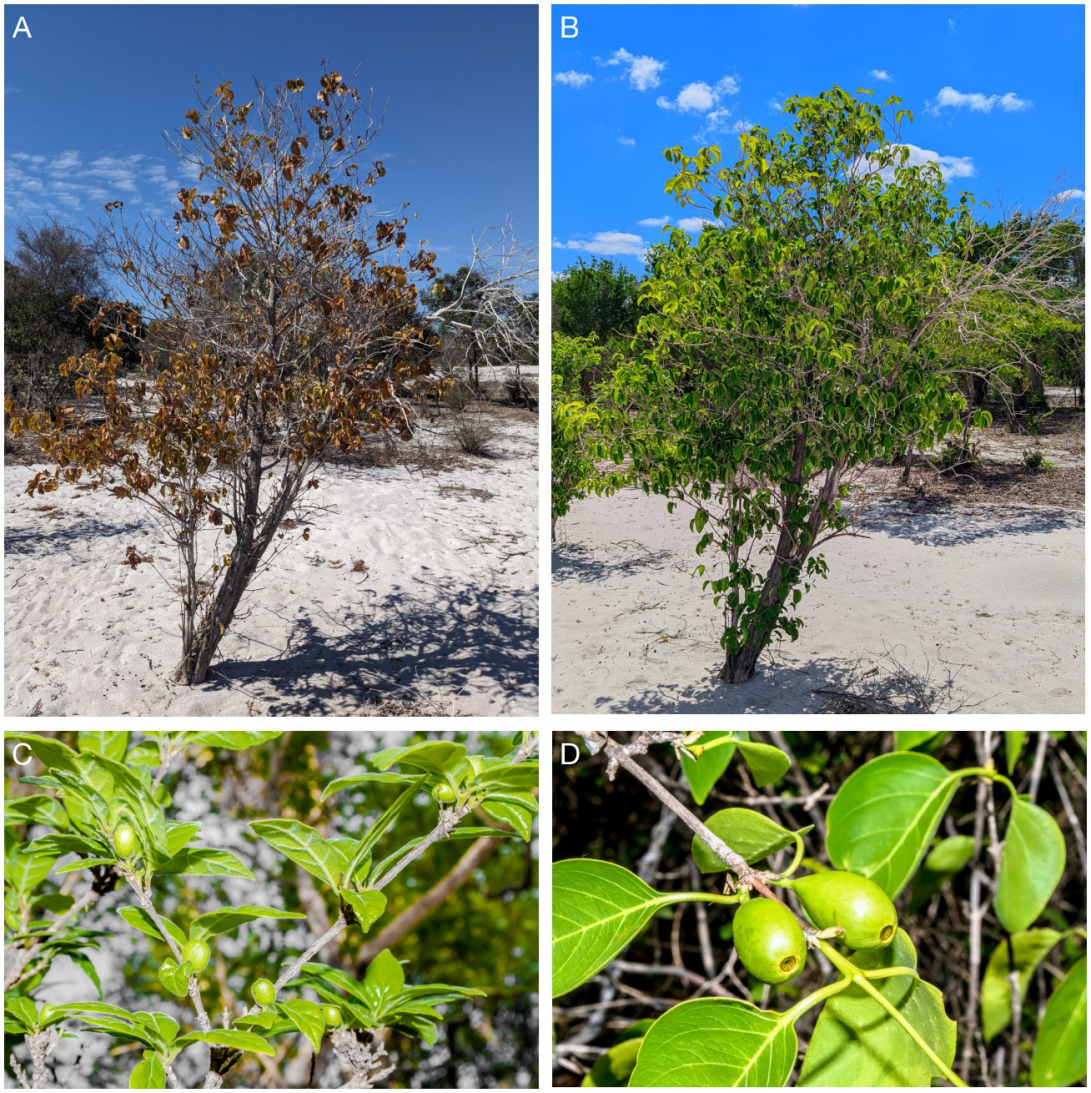
Pictures of Baracoffea. A. *Coffea ambongensis* in its natural environment during the dry season. B. *Coffea ambongensis* in its natural environment during the wet season. C. *Coffea bissetiae*, fruits. D. *Coffea boinensis*, fruits. Pictures: Rickarlos Bezandry.

In the *Coffea* genus, 66 species are native to the Indian Ocean Comoros Archipelago and Madagascar [10, 15, 18]. These species were initially classified into 8 botanical series, then reorganized into 6 series on the basis of leaf, flower and fruit characteristics: Verae Chev, Multiflorae Chev, Subterminal (ex Terminal Chev), Garninoïdes Chev, Millotii complex, and Humblotianae Ler.-Mauritianae Chev [19]. Similarly to the extraordinary biodiversity of Madagascar organisms [20], *Coffea* species have colonized a variety of environments and show a huge phenotypical diversity [18]. Recent phylogenetic analyses have proposed a late diversification of species in Madagascar (~ 8 Mya), which occupy a large part of the territory [11, 21]. These include a group of nine species renamed the “Baracoffea” alliance by Davis and Rakotonasolo [22] (*Coffea ambongensis* J.-F.Leroy ex A.P.Davis & Rakotonas, *C*. *bissetiae* A.P.Davis & Rakotonas, *C*. *boinensis* A.P.Davis & Rakotonas, *C*. *labatii* A.P.Davis & Rakotonas., *C*. *namorokensis* A.P.Davis & Rakotonas., *C*. *pterocarpa* A.P.Davis & Rakotonas, *C*. *decaryana* J.-F.Leroy, *C*. *grevei* Drake ex A.Chev., *C*. *humbertii* J.-F.Leroy). The later clade shows unusual ecological habits in that they occupy the sandy soils of the dry deciduous forests of western Madagascar. They grow and survive in regions with a hot and dry climate [22] but are mostly threatened [23] by habitat loss (Fig 1). Morphologically, these species also share singular characters for coffee trees, such as terminal inflorescence, sympodial development, and deciduous leaves, among others [22]. Although Baracoffea species might hold key traits (morphological or physiological) explaining their tolerance to considerably drier and hotter climates in contrast to their sister clade *Coffea*, only few studies have attempted to interpret the Baracoffea phylogeny. Understanding their evolutionary history, and identifying the driving factors behind their recent diversification, holds the potential to reveal novel traits suitable for breeding practice, thereby improving drought tolerance of coffee tree cultivated varieties. Using gene and intergenic sequence data of chloroplast and nuclear origin, Maurin and coworkers concluded on Baracoffea monophyly with the analysis of seven species, while the approach they employed did not allow for identification of related *Coffea* species [24]. More recently, Hamon and coworkers (2017) used a GBS (Genotyping by Sequencing) approach with 28,800 SNP (Single Nucleotide Polymorphism) nuclear markers and confirmed Baracoffea monophyly, but this study only considered two species: *C*. *labatii* and *C*. *humbertii* [11]. Although the monophyly of the Baracoffea group is likely, additional analyses are required to ascertain the relationship between Baracoffea related *Coffea* species, more specifically with regard to understanding their evolutionary history and how it contributed to the adaptation of these species.

In this contribution, we aim to resolve the chloroplast and nuclear phylogenetic analyses of three Baracoffea species: *C*. *ambongensis*, *C*. *bissetiae* and *C*. *boinensis*, from the Boeny region of Madagascar. These three species are the only Baracoffea among nine species that can be studied currently, thanks to on-going research at the University of Mahajanga, Madagascar. Using the obtained phylogenies coupled with genome size, bioclimatic data of Madagascar and principal component analysis, we test whether climate has been responsible for the evolutionary trajectory of Baracoffea species.

## Materials and methods

### Plant material

Information on the origin and geographical occurrences of the three species: *Coffea ambongensis* J.-F.Leroy ex A.P.Davis & Rakotonas, *C*. *boinensis* A.P.Davis & Rakotonas. And *C*. *bissetiae* A.P.Davis & Rakotonas and available below have been obtained from Rickarlos Bezandry: *Coffea ambongensis*, code BR071, collected in the Antsanitia forest (S16°17’45.2; E046°49’36.4); *C*. *boinensis*, code BR051 collected in Ankarafantsika National Park (S16° 00’ -S16° 19’; E46° 34’–E47° 17’) and *C*. *bissetiae*, code BR031 collected in the Ankarafantsika region. This study also includes GPS coordinates retrieved from [18, 25]. Additional occurrences were extracted from [19]. Plant collection permits have been obtained from Madagascar’s Ministry of the Environment and Sustainable Development (Department of Protected Areas, Renewable Natural Resources and Ecosystems).

### Illumina sequencing

Dry leaves from all three species were used for DNA extraction using the Dneasy^®^ plant mini kit protocol (Qiagen, France). DNA was sequenced by Eurofins, France using the Illumina platform in paired-end 150-base sequencing according to the manufacturer’s instructions. For *Coffea ambongensis*, 2X49 million raw reads (representing 14.7 Gb), for *C*. *boinensis*, 2X58 million (17.5 Gb) and for *C*. *bissetiae*, 2X46 million (13.8 Gb) were produced (BioProject PRJNA898910).

### Plastid genome reconstruction and comparison and nuclear SNP calling

*De novo* reconstruction of the plastid genome was carried out following the protocol of Charr and colleagues [21]. The newly assembled plastid sequences obtained in this study are annotated and deposited at NCBI (accessions: ON101707, ON101708 and ON117418). These three whole chloroplast genomes were compared with the *C*. *arabica* (EF044213) chloroplast using the online genome analysis program mVISTA [26], and the LAGAN global multiple alignment. Illumina reads were mapped against the *Coffea canephora* reference genome [27] using Bowtie2 [28] and default parameters. Then, to compare the three Baracoffea species against the *Coffea* genus, the SNP (Single Nucleotide Polymorphisms) markers previously developed by Hamon and coworkers [11] via a GBS (Genotyping By Sequencing) were called using NGSEP [29] MultisampleVariantsDetector. A total of 28,800 SNPs were retrieved for *C*. *ambongensis*, *C*. *boinensis* and *C*. *bissetiae* and merged with the previous GBS database. This database was filtered to include only Malagasy species (S1 File), as well as filter out the fixed variants among the species, variants with minimum allele frequency >< 0.01, and multiallelic sites. A Principal Component Analysis (PCA), a distance matrix and a Neighbor-Joining reconstruction of the species was performed using the final SNP database.

### Phylogenetic analyses

Maternal phylogeny was reconstructed following [21]. The three Baracoffea chloroplast sequences were aligned with the full-length chloroplast sequences of *Coffea* species obtained in Charr et al. 2020 using Mafft [30]. A total of 57 full-length chloroplast sequences were aligned and analyzed using RaxML ng Version 0.9 [28] with the same parameters used as in Charr and coworkers [21], with 100 repetitions (bootstraps). *Empogona congesta* (ex *Tricalysia congesta*), a Rubiaceae species, was used as an outgroup. All chloroplast sequences are accessible on NCBI (accessions available in Charr and coworkers work).

To complete the maternal phylogenetic analysis, a nuclear phylogenetic analysis was conducted using the SNP database. Similarly to Charr and colleagues, the 28,800 SNPs were aligned with Mafft and used for Maximum Likelihood phylogenetic analysis with RaxML ng Version 0.9 with the same parameters used as in Guyeux and colleagues (2019) [31] (General Time Reversible (GTR) model of nucleotide substitution under the Gamma model of rate heterogeneity). The tree was constructed with 40 species of Malagasy origin, one species from Mayotte (*C*. *humblotiana*), three species from the Mascarene Islands, one species from East Africa and one outgroup (*Empogona congesta*, Rubiaceae), with 100 bootstraps (S1 File). The SNPs used in this study are available in concatenated sequence in FASTA format (S2 File). Phylogenetic trees were edited with Itol, https://itol.embl.de).

### Estimation of nuclear DNA content

Nuclear DNA content was measured by flow cytometry at the Imagif Cell Biology platform (Gif-sur-Yvette, France) according to Razafinarivo and coworkers [25]. Measurements (presented in pg content per 2C) were performed for *C*. *ambongensis* BR071, *C*. *boinensis* BR051 and for *C*. *bissetiae* BR03. Nuclear DNA content for 35 Malagasy species were extracted from Razafinarivo and coworkers [25].

### Environmental parameters and statistical analysis

Climatic data were extracted from the Global Positioning System coordinates of each species (S1 File) and from bioclimatic variables were extracted from WorldClim (http://www.worldclim.org) with a spatial resolution of 10 arcmin. QGIS (version 3.16) was used to extract corresponding values for each variable selected. A principal component analysis (PCA) was made using packages ‘FactoMineR’ and ‘factoextra’ from R in RStudio version 2023.6.0.421. The PCA was carried out based on 30 environmental and climatic variables extracted from Worldclim (http://www.worldclim.org; 19 variables) and Madaclim (https://madaclim.cirad.fr/; 21 variables) and nuclear DNA estimation. After PCA analysis, seven variables were kept as follow: annual climatic water deficit (mm, WatDeficit), number of dry months (DryMonths), annual temperature (°C, MeanTemp), temperature seasonality (°C, TempSeas), mean annual precipitation (mm, AnnPrecip), altitude (m, Alt), and the DNA nuclear estimation (2C, X2C). Data were plotted according to the botanical series of species (including Baracoffea, S1 File). Species for which genome sizes were not available were removed from further analysis (S1 File). Linear regressions were done using packages ‘tidiverse’ and ‘car’ from R studio and using climatic variables ang GPS positions. To include the genomic variants in the analysis, a PCA was also made using ‘FactoMineR’ and ‘factoextra’ packages. The PCA was carried out with the five principal components from the genomic variants analysis, the seven environmental variables previously retained, and the genome size. Data was plotted using the same criteria as the environmental PCA. Finally, a correlation was performed between each pair of variables using the chart.Correlation function from the PerformanceAnalytics R package.

## Results

### Maternal phylogenetic analysis

**Fig 2:**
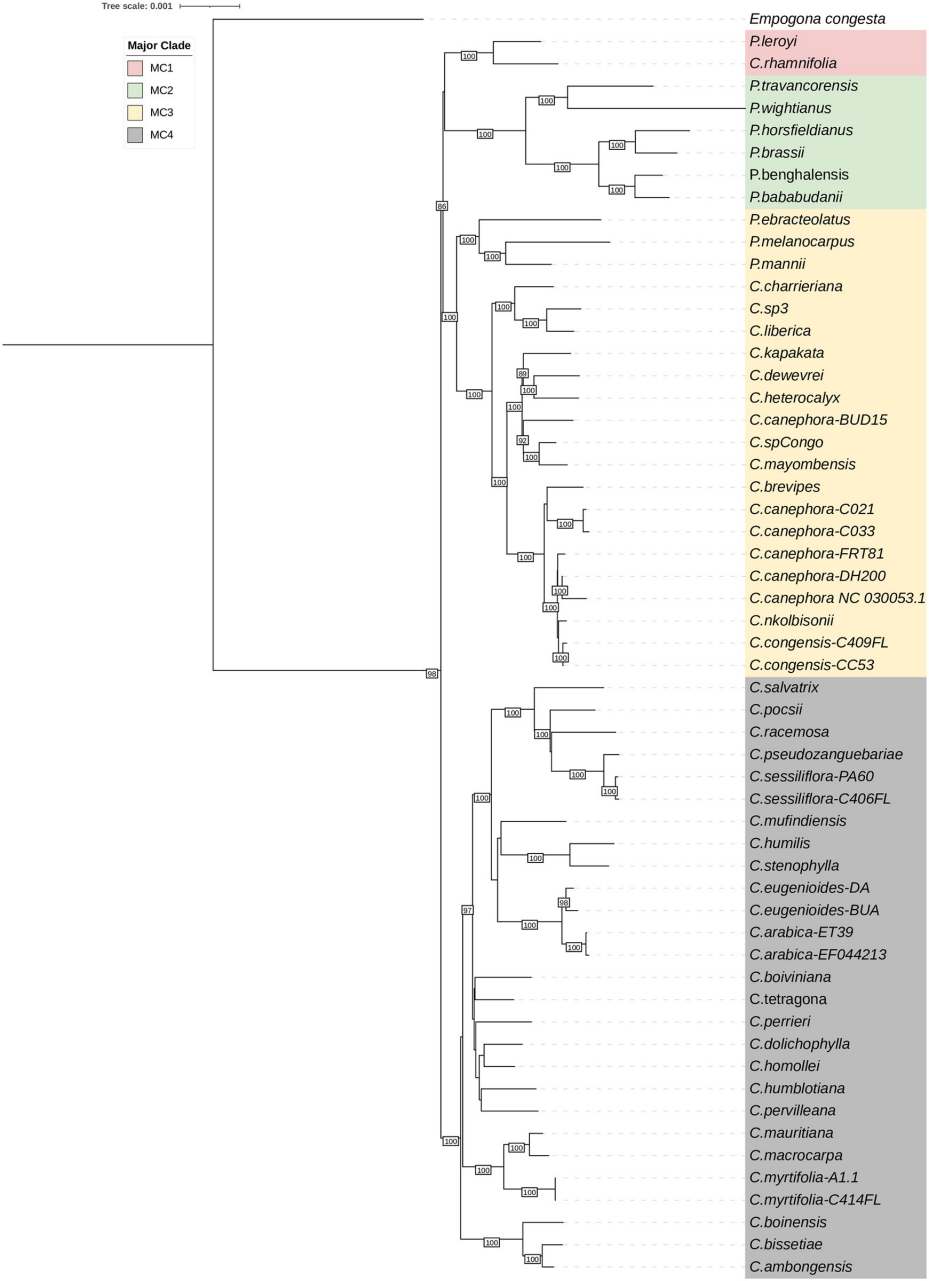
Maximum likelihood tree of 57 full-length chloroplast genomes of the genus *Coffea*. Major clades (MC1-MC4) are color-coded in a manner similar to the publication in Charr et al., 2020. Only bootstrap values greater than 85 are shown here. Bootstrap value = 100.

The chloroplast genome sequences of *Coffea ambongensis* (ON101708), *C*. *boinensis* (ON101707) and *C*. *bissetiae* (ON117418) were reconstructed in their entirety using NOVOPlasty software [32], based on full Illumina DNA sequencing. They have a length of 154.826 bp, 154.879 bp and 154.781 bp, for *C*. *ambongensis*, *C*. *boinensis* and *C*. *bissetiae*, respectively (S3 File). These sequences were fully aligned with the whole chloroplast genome sequences of one outgroup species (*Empogona congesta*, Rubiaceae), ten species formerly classified in the genus *Psilanthus* and 43 species of *Coffea*, whose sequences have recently been published [21] and are available in NCBI public databases. The maximum likelihood tree reveals four major chloroplast clades (MC1 to MC4) as previously established. The chloroplast genomes of Baracoffea are found in the MC4 clade (in gray, Fig 2) comprising species from the Indian Ocean islands, *Coffea* species from East Africa, two *Coffea* from West Africa (such as *C*. *stenophylla* and *C*. *humilis*) and species from Central and East Africa (including *C*. *arabica*). Within this clade, Baracoffea species are grouped into a monophyletic group (100% bootstrap support) hereafter referred to as the Baracoffea clade. The previously retrieved Baracoffea clade appears basal in the MC4 chloroplast group. It first diverged from the other MC4 species originating from various geographical regions, including Comoros archipelagos and Indian Ocean Islands (*C*. *humblotiana*, *C*. *myrtifolia*, *C*. *mauritiana and C*. *macrocarpa*), which then diverged from other Madagascar species (*C*. *tetragona*, *C*. *boiviniana*, *C*. *perrieri*, *C*. *dolichophylla*, *C*. *homollei*, *C*. *pervilleana*) (Fig 2). The three chloroplast genomes of Baracoffea were compared and plotted against *C*. *arabica* as reference using mVISTA (S3 File). Higher sequence variation is observed in conserved non-coding regions than in conserved protein-coding regions. However, some small variations common for the three Baracoffea could be observed in *rpoC1*, *clpP petD* and *ycf2* genes (S4 File).

### Nuclear phylogenetic analysis

**Fig 3:**
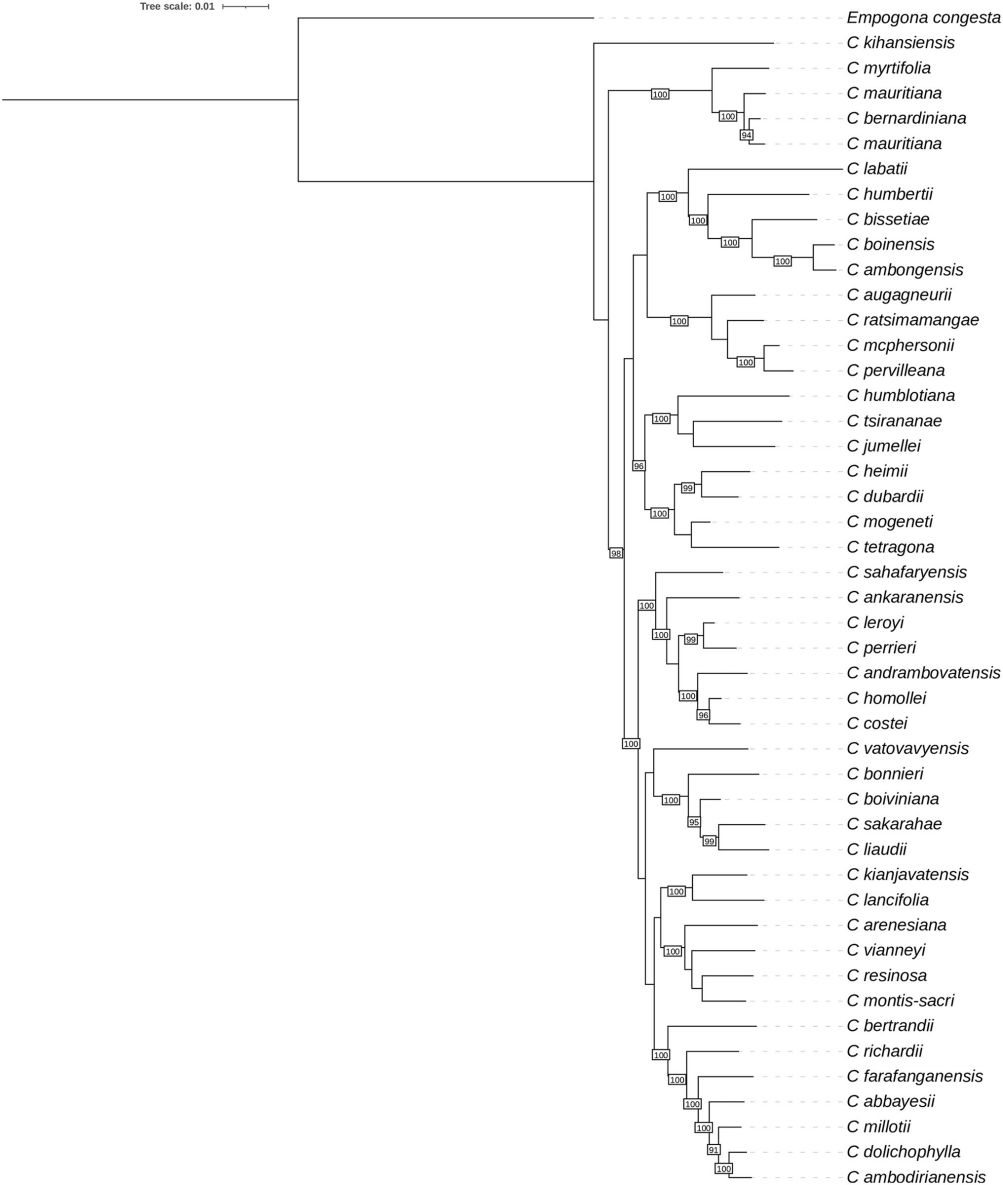
Maximum likelihood tree with 28,800 concatenated nuclear SNP markers for 47 species. Only bootstrap values above 85 are shown in the tree. Bootstrap value = 100.

In the Baracoffea group, five species are represented as molecular data in databases: *C*. *ambongensis*, *C*. *boinensis* and *C*. *bissetiae* (this analysis) and *C*. *humbertii* and *C*. *labatii* [11]. As with maternal phylogeny, nuclear tree analysis supports the monophyly of Baracoffea clade within species of Malagasy origin (100% bootstrap support). This clade is sister to a group of species classified in the botanical series ’Subterminal’ (*Coffea augagneurii*, *C*. *pervilleana*, *C*. *ratsimamangae* and *C*. *mcphersonii* (synonym of *C*. *vohemarensis* in this work). Other species classified in this series are scattered in other clades of the tree (*C*. *boiviniana*, *C*. *vatovavyensis*, *C*. *bonnieri*, *C*. *tsirananae*, *C*. *jumellei*, *C*. *sakarahae*) (Fig 3; S1 File).

### Baracoffea genetic diversity

**Fig 4:**
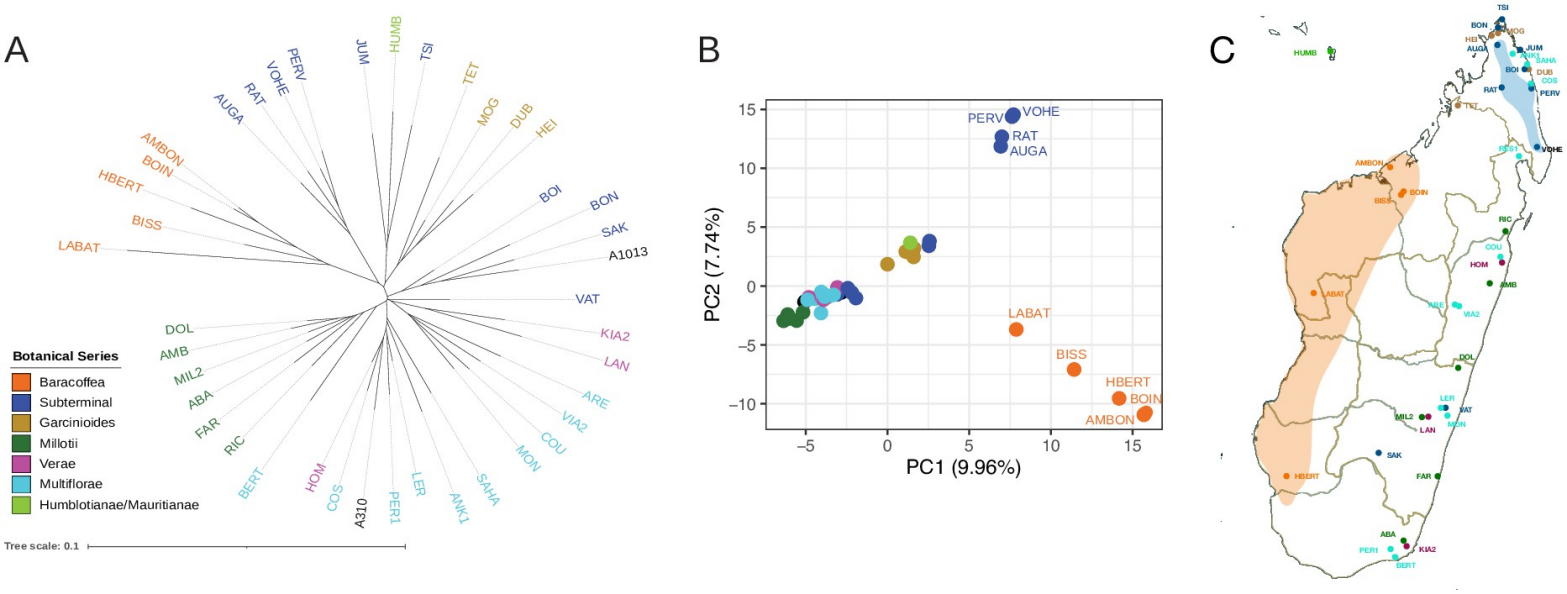
Baracoffea genetic diversity assessed using 9,665 variant SNPs. A. Genetic distance of *Coffea* species from Madagascar. B. PCA analysis. C. Geographic position of species used in the analysis.

A total of 28,800 variants were retrieved for C. ambongensis, C. boinensis and C. bissetiae from the Illumina datasets, with less than 2% of missing data for each species. This variation database was merged with the GBS panel [11], including only the Malagasy species. The final variation database (filtered by MAF > 0.01 and biallelic SNP) was composed of 9,665 variants. The percentage of missing data of this database was 5.95%. The mean heterozygosity of the species was 2.65%, and the Baracoffea species showed between 1.3 and 3.2% heterozygotic sites (See S5 File for details). The genetic distances among the species were calculated and are shown in Fig 4A, where a cluster of Baracoffea species is differentiated from the other species. This result is consistent with the PCA (Fig 4B), where the first component separates Baracoffea species from species in the Garcinioïdes, Millotii, Multiflorae, Verae series, and the majority of Subterminal; and second component separates the group of Subterminal species most closely related to Baracoffea (Fig 4C).

### Relationships to geographical, bioclimatic and environmental data in Madagascar

**Fig 5:**
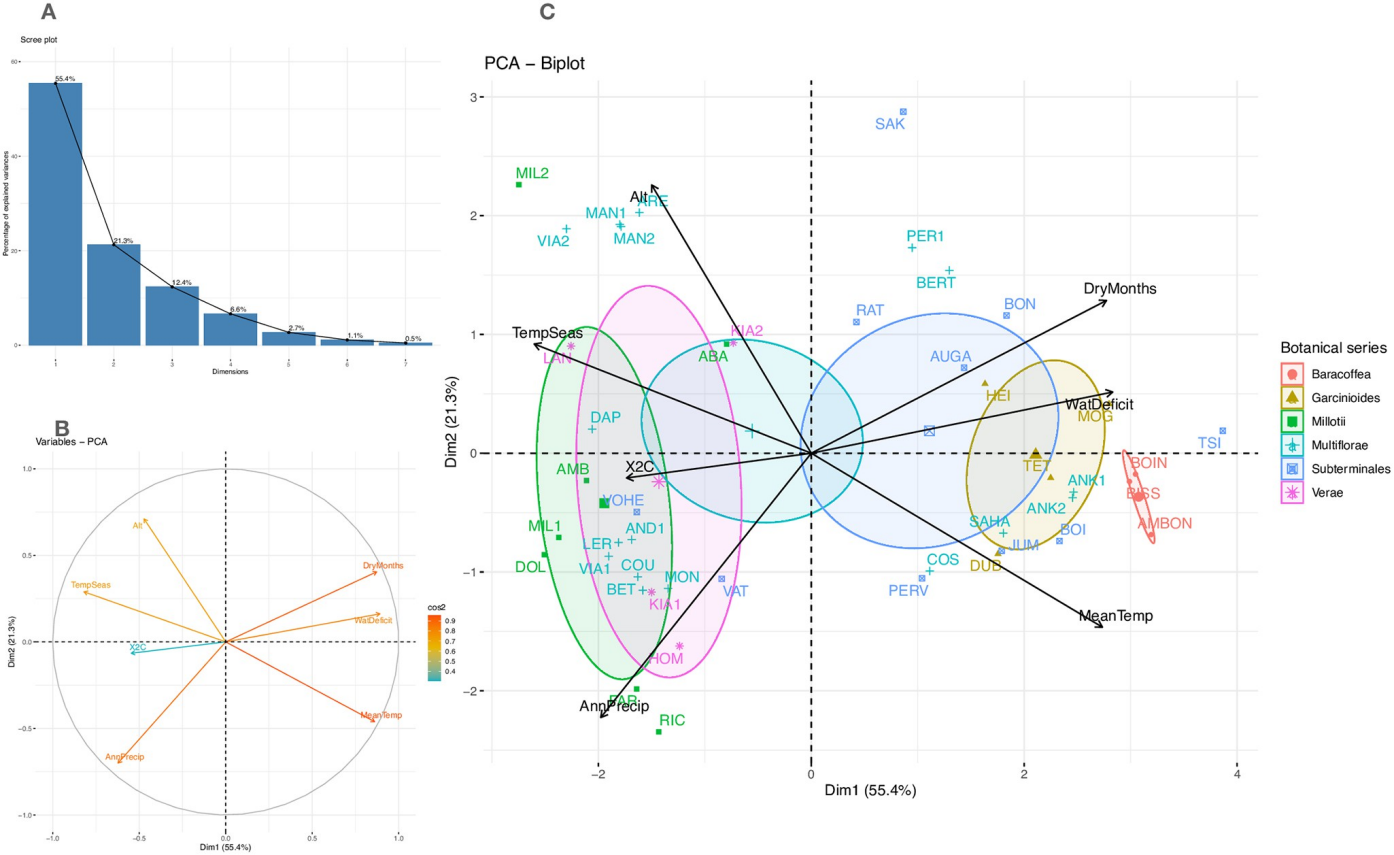
Principal component analysis with eight quantitative variables (climate, geography and genomics), botanical series (including Baracoffea) and Malagasy Coffea species (species codes are explained in S1 File). AnnPrecip: Annual precipitation, TempSeas: Temperature seasonality, MeanTemp: Mean temperature, X2C: Genome size (2C, pg), Alt: Altitude, WatDeficit: Water deficit, DryMonths: number of dry months. A. Scree plot. B. Variables of PCA. C. Biplot.

To study in detail the relationships between the evolution of Malagasy coffee species and their climatic environment in their area of origin, we compared our nuclear phylogeny with 30 environmental and climatic variables (see Material and methods) extracted from their geographical occurrences. Among the 30 variables, seven were kept after correlation and PCA analyses: annual climatic water deficit (mm, WatDeficit), number of dry months (DryMonths), annual temperature (°C, MeanTemp), temperature seasonality (°C, TempSeas), mean annual precipitation (mm, AnnPrecip), altitude (m, Alt). In addition, DNA nuclear estimation is also used (genome size, 2C, X2C) (S6 File). The Principal Component Analysis based solely on these seven climate variables shows that more than half of the variance is explained in dimension 1 (55.4%), and almost 76% in two dimensions (Fig 5A). The correlation circle (Fig 5B) shows an opposite correlation between annual precipitation and the number of dry months, between genome size and water deficit, and a positive correlation between the number of dry months and water deficit. The biplot (Fig 5C) superimposing the correlation circle and the species belonging to the botanical series, clearly shows the associations of species with specific environmental characteristics. The Baracoffea clade is clearly correlated with elevated annual temperatures, water deficit and number of dry months. The Multiflorae and Subterminal botanical series are the most diverse with respect to environmental parameters, suggesting contrasting adaptations for the species of these botanical series. The clade comprising four species from the Subterminal series (*C*. *augagneurii* (AUGA), *C*. *pervilleana* (PERV), *C*. *ratsimamangae* (RAT) and *C*. *mcphersonii* (synonym of *C*. *vohemarensis*, VOHE)) is scattered throughout the biplot. Also noteworthy are the series of the Millotii complex and Verae, which are found at the opposite end of the Baracoffea clade, with climatic parameters strongly correlated with high annual rainfall. Interestingly, genome size (2C) is relatively correlated with annual precipitation and opposite to the number of dry months and temperatures. Observations made between genome sizes and environmental variables were also analyzed using linear regressions (S7 File). These regressions indicate a strong negative correlation between genome size and geographical latitudes, and number of dry months and a positive correlation with the temperature seasonality (p < 0.001) and to a lesser extent a negative correlation with the mean temperature (p < 0.005), water deficit (p < 0.05) and a positive correlation with annual precipitations (p < 0.05) (S7 File).

### Relationships between genetic diversity and climatic variables

**Fig 6:**
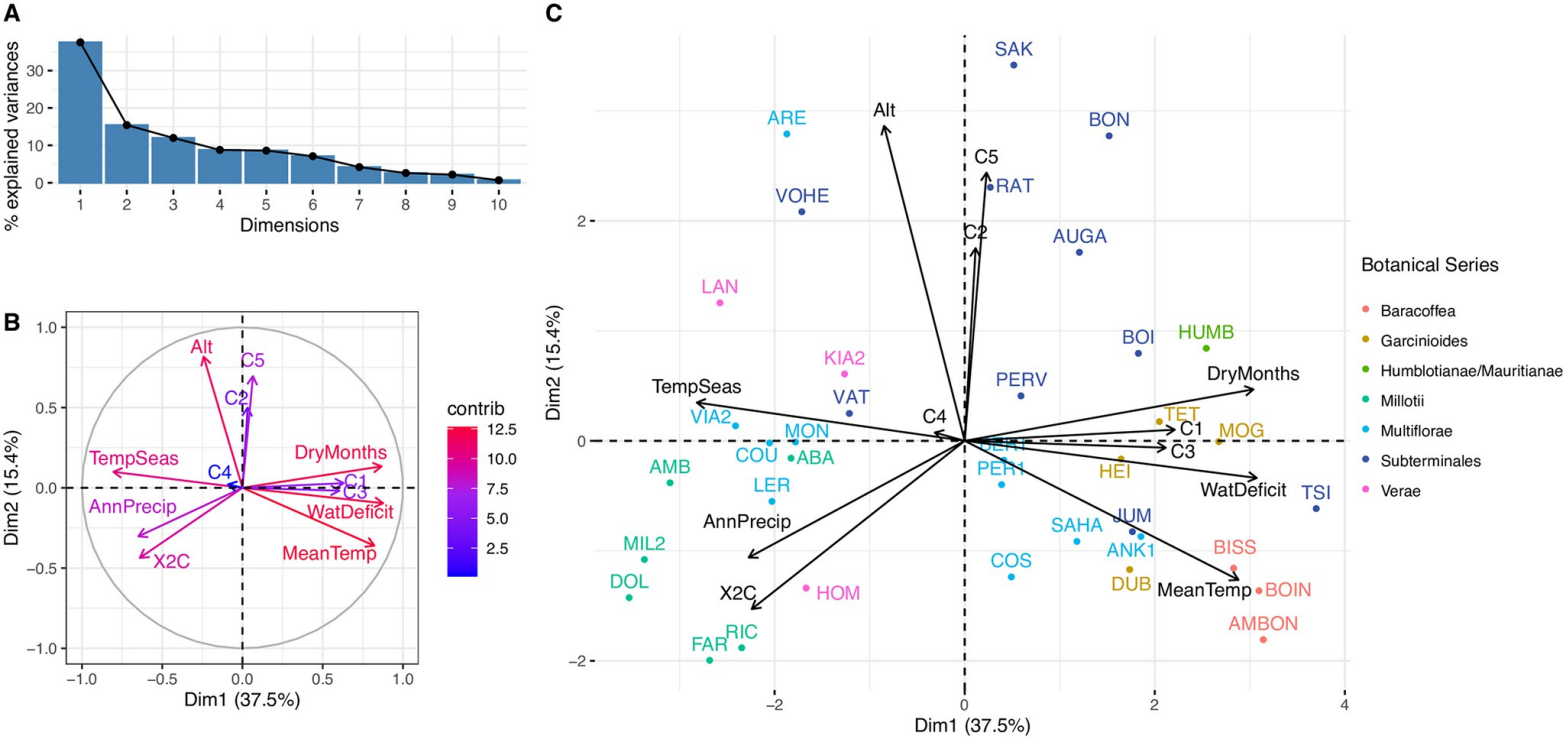
Principal component analysis with seven quantitative variables (climate, geography and genomics) and the five principal components from biallelic SNP. Botanical series and *Coffea* species are shown (species codes details in S1 File). AnnPrecip: Annual precipitation, TempSeas: Temperature seasonality, MeanTemp: Mean temperature, X2C: Genome size (2C, pg), Alt: Altitude, WatDeficit: Water deficit, DryMonths: number of dry months. A. Scree plot. B. Variables of PCA. C. Biplot.

To establish a preliminary relationship between the genomic variants and the environmental variables, a PCA was calculated. Genomic variants were represented as the five principal components generated (C1 to C5) with the seven environmental variables previously retained and the genome size. The resulting first component explained the 37.5% of the variance, whereas the second one explained 15.4% (Fig 6A). In Fig 6B, the contribution of each variable is represented, and it shows that C1 and C3 were associated with the first component, and are possibly correlated with the Mean Temperature, Dry Months, Temperature seasonality, Annual precipitation, and Water Deficit. On the other hand, C2 and C5 were related to the second component and could be correlated with the Altitude. Finally, Fig 6C shows a clear differentiation of the Baracoffea species when including not only the genomic variants nor the environmental characteristics, but the combination of both factors. Therefore, a correlation between each pair of variables was computed to identify significant ones. The results showed a significant positive correlation between C1 and the Water deficit and Mean temperature (p-value < 0.001), whereas a significant negative correlation when comparing with the Sea temperature (p-value < 0.001). C3 reported similar correlations to C1; however, it showed a higher correlation with the Genome Size (p-value < 0.01). C2 and C5 showed a significant correlation with the altitude (p-value <0.05); but the correlation coefficient was not strong (See S8 File for details). These results suggest an association between the environmental and genomic variants; however, to identify the specific traits (SNP, gene, regions) associated with each environmental variable, a sampling of multiple individuals per species is required.

## Discussion

To understand the evolution of species and the relationship between genetic diversity and adaptation, genomics has become an essential tool, thanks to the availability of "second- and third-generation" short- or long-read sequencing solutions and powerful bioinformatics tools, enabling the assembly of chloroplast genomes or the identification of numerous markers such as SNPs. These approaches are rapid and robust, and have demonstrated enormous potential for diversity analysis, particularly in the gene pools present in "Crop Wild Relatives", and for the improvement of cultivated species [33, 34]. Indeed, Crop Wild Relatives are known to be able to mitigate the impact of climate change because their genetic composition confers greater tolerance to drought and other abiotic and biotic stresses [35]. Recently, genomic approaches have provided fundamental results in the genus *Coffea*, enabling the complete and robust resolution of nuclear phylogeny [11] and the identification of major chloroplast clades [21]. The resolution of this approach also enabled the identification of unexpected diversity in the *C*. *canephora* species. The power of this approach also lies in the possibility of repeatedly including new genomic material to clarify the evolutionary history of a particular clade.

In this study, we carried out a phylogenetic analysis based on chloroplast and nuclear sequence data obtained by Illumina sequencing, in order to elucidate the evolutionary history of the Baracoffea group, by first, ascertaining the monophyly of the group based on three Baracoffea species: *C*. *ambongensis*, *C*. *boinensis*, and *C*. *bissetiae* from the Boeny region, Madagascar. Our results robustly assign these three species to a monophyletic group, whether using maternal or nuclear phylogeny. In the maternal phylogeny, this group is part of the major clade (MC4) like the other Malagasy species and some African species, but the analysis does not allow us to place with confidence the clade in relation to either Malagasy or Mascarene species. Indeed, the bootstrap value of the branch is not statistically significant (59%) to conclude definitively on their evolutionary relationship with the other species of the MC4 clade, and more molecular data, specifically retrieved from a complete sampling of Baracoffea species will be required conclude on the position of Baracoffea species in relation to its relatives. Indeed, in the nuclear phylogeny this group is included in the Malagasy *Coffea*, consistently with Hamon and coworkers [11] for two other species (C. *humbertii and C*. *labatii*). Our analysis also confirms the close relationship of the Baracoffea clade with a clade composed of four species from the Subterminal botanical series. This relationship put this clade as a very suitable model for studying species adaptation and evolution, as Baracoffea developed, and likely diversified, in dry deciduous forests of western Madagascar, whereas the four species from the Subterminal Serie of the sister clade (i.e. *C*. *auganieurii*, *C*. *pervilleana*, *C*. *ratsimamangae and C*. *mcphersonii*) originate from northern Madagascar with more heterogeneous environments (dry region, but containing remnants of humid forests and monsoon rainforests and bordered by zones with very distinct climates (sub-humid, humid and mountain) [25]). The molecular dating performed by Hamon and coworkers using the externally-calibrated dates estimated for the divergence between *Coffea* and *Psilanthus* [36], indicates a divergence estimate of 7 My between Baracoffea and this sister clade, suggesting a diversification that followed the divergence between African and Indian Ocean islands species (>10 My) and the divergence between Malagasy and Mascarene species.

Madagascar has a high biodiversity that evolved in isolation, characterized by environmental gradients, common patterns of micro-endemism between taxa and numerous evolutionary radiations [37]. Madagascar, in particular, displays disparate and contrasting biomes, ranging from humid tropical climates (East) to sub-arid climates (South), including the dry deciduous forests of the West. These contrasting climates, corresponding to large bioclimatic zones, have undoubtedly contributed to the diversification and adaptation of species on the island. This information indicates that the Baracoffea clade has diversified and adapted uniformly to the dry climate of western Madagascar, whereas its sister clade has undergone contrasting adaptations. However, we don’t know how the climate varied during the speciation of the Baracoffea 7 million years ago, but a recent report suggested a great monsoon variability over the last 5 million years, which would have favored the evolution of Madagascar’s flora [38]. Phylogenetic analysis coupled with bioclimatic analysis suggests an adaptive speciation for all coffee species in Madagascar, with climate playing a predominant role. Combining genetic and environmental data, it has become clear that the Baracoffea group and the phylogenetically closest Subterminal group of species have differentiated under different climatic constraints and adapted to different ecological niches. Future analyses between populations of different species will probably enable us to identify the markers under selection and therefore the associated genes involved in these adaptations.

Plant species evolution and adaptation to various environmental drivers can additionally result in variations in genome size. Apart from polyploidy, these variations are linked to the number of copies of transposable elements in the nuclear genome [39]. Razafinarivo and coworkers [25], noted the presence of a geographical gradient of *Coffea* genome size variation in Africa and the Indian Ocean islands. In Madagascar, this gradient increases from north-east to south-west. Our analysis confirms the geographical variations observed by Razafinarivo and coworkers and suggests a relationship between the genome size of Madagascar’s coffee species and bioclimatic parameters, specifically aridity and temperature. In Brassicaceae, a similar correlation between genome size and seasonal climate was identified, suggesting a possible role for genome size in adaptation to various climatic constraints [40]. Better still, a negative correlation between genome size and aridity [41] has been demonstrated in palms and seems to be associated with the copy number of transposable elements such as LTR retrotransposons. The sequencing of the nuclear genomes of Madagascar’s coffee species could provide answers to these size variations and the involvement of transposable elements and their impact on genomes.

In order to address the emerging challenges posed by global climate change, further studies should focus on improving our understanding of how genome size could directly improve adaptability of crops, including coffee tree varieties. Our study highlights the need for conducting additional studies to improve our comprehension of the relation between genome size and climatic constraints tolerance, of how these climatic constraints likely played a role of driver on species diversification and what could be the potential morphological and physiological traits that can be related to both genome size and abiotic constraint overcoming capacity. Answering these questions are of critical significance to improve breeding practices and face current climatic challenges.

## Supporting information

S1 File Table with species names, gps positions, genome size and climatic data used. (XLSX)

S2 File Concatenated nuclear SNP sequences in fasta format. (FASTA)

S3 File Graphical maps of C. ambongensis, C. boinensis and C. bissetiae chloroplasts performed with OGDRAW (https://chlorobox.mpimp-golm.mpg.de/OGDraw.html). (PDF)

S4 File mVISTA alignments of chloroplast genomes of *C*. *ambongensis*, *C*. *boinensis*, *C*. *bissetiae* and *C*. *arabica*. (PDF)

S5 File Number of genotyped sites, % of missing data and % of heterozygotic sites. (XLSX)

S6 File Correlation data between climatic variables. (PDF)

S7 File Linear regression analyses between genome size and environmental data. (PDF)

S8 File Correlation data between climatic and genetic variables. (PDF)
