# Supplementary material for: The evolutionary history of three Baracoffea species from western Madagascar revealed by chloroplast and nuclear genomes
Source: PLoS One. 2024 Jan 11;19(1):e0296362. doi: 10.1371/journal.pone.0296362 (PMC10783717; doi:10.1371/journal.pone.0296362)
Supplement: S3 File — (PDF) [file pone.0296362.s003.pdf]

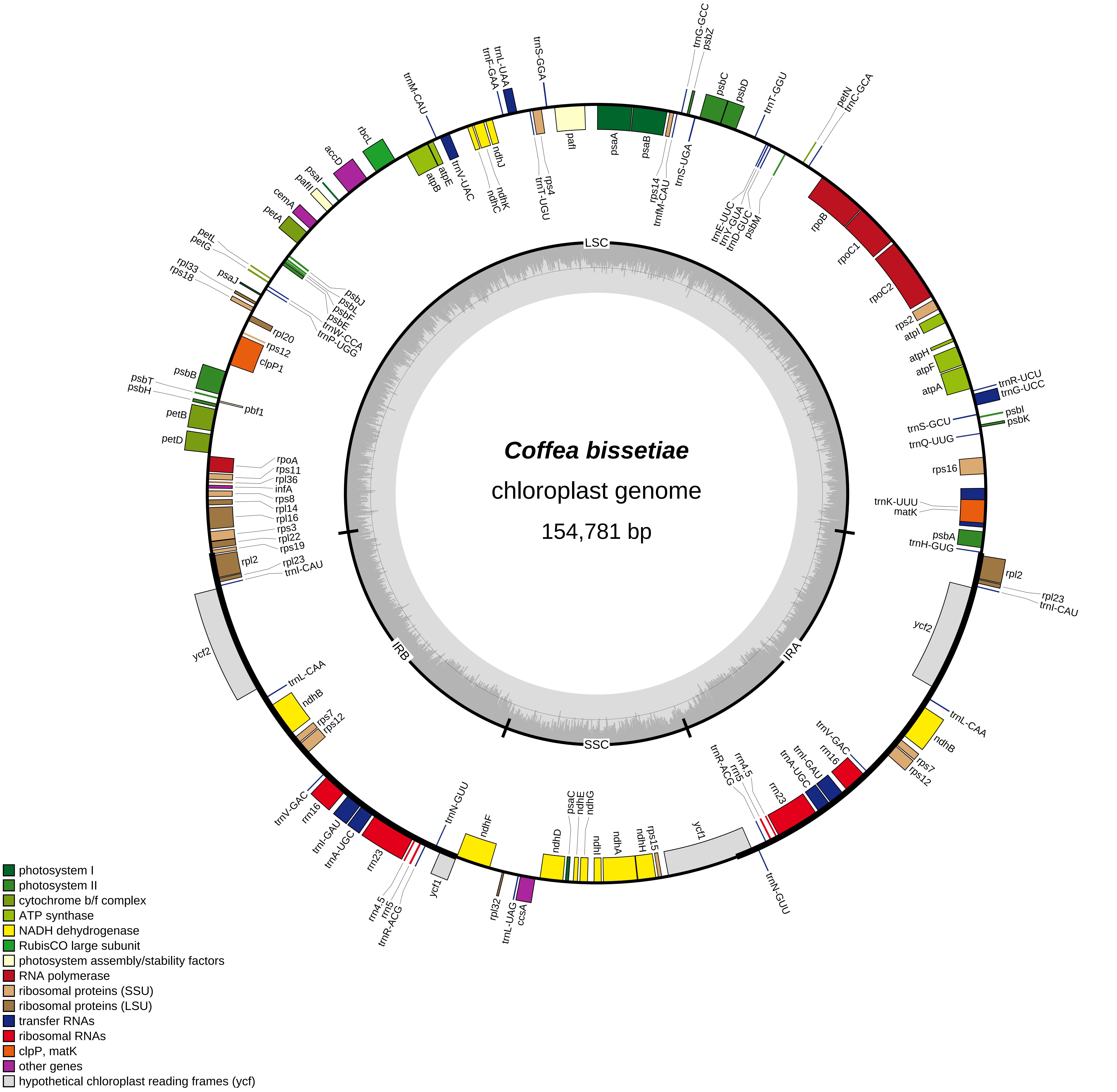

Coffea bissetiae
chloroplast genome
154,781 bp
LSC
IRA
SSC
IRB
trnG-GCC
psbZ
psbC
psbD
trnT-GGU
petN
trnC-GCA
trnS-GGA
trnL-UAA
trnF-GAA
trnM-CAU
pafI
psaA
psaB
rps4
trnT-UGU
ndhJ
ndhK
ndhC
trnV-UAC
atpE
atpB
rbcL
accD
psaI
pafII
cemA
petA
rps14
trnfM-CAU
trnS-UGA
trnE-UUC
trnY-GUA
trnD-GUC
psbM
rpoB
rpoC1
rpoC2
rps2
atpI
atpH
atpF
atpA
trnR-UCU
trnG-UCC
psbI
psbK
trnS-GCU
trnQ-UUG
rps16
trnK-UUU
matK
psbA
trnH-GUG
rpl2
rpl23
trnI-CAU
ycf2
trnL-CAA
ndhB
rps7
rps12
trnV-GAC
rrn16
trnI-GAU
trnA-UGC
rrn23
rrn4.5
rrn5
trnR-ACG
trnN-GUU
ycf1
rps15
ndhH
ndhA
ndhI
ndhG
ndhE
psaC
ndhD
ccsA
trnL-UAG
rpl32
ndhF
petL
petG
psbJ
psbL
psbF
psbE
trnW-CCA
trnP-UGG
rpl33
rps18
psaJ
rpl20
clpP1
psbB
psbT
psbH
pbf1
petB
petD
rpoA
rps11
rpl36
infA
rps8
rpl14
rpl16
rps3
rpl22
rps19
photosystem I
photosystem II
cytochrome b/f complex
ATP synthase
NADH dehydrogenase
RubisCO large subunit
photosystem assembly/stability factors
RNA polymerase
ribosomal proteins (SSU)
ribosomal proteins (LSU)
transfer RNAs
ribosomal RNAs
clpP, matK
other genes
hypothetical chloroplast reading frames (ycf)

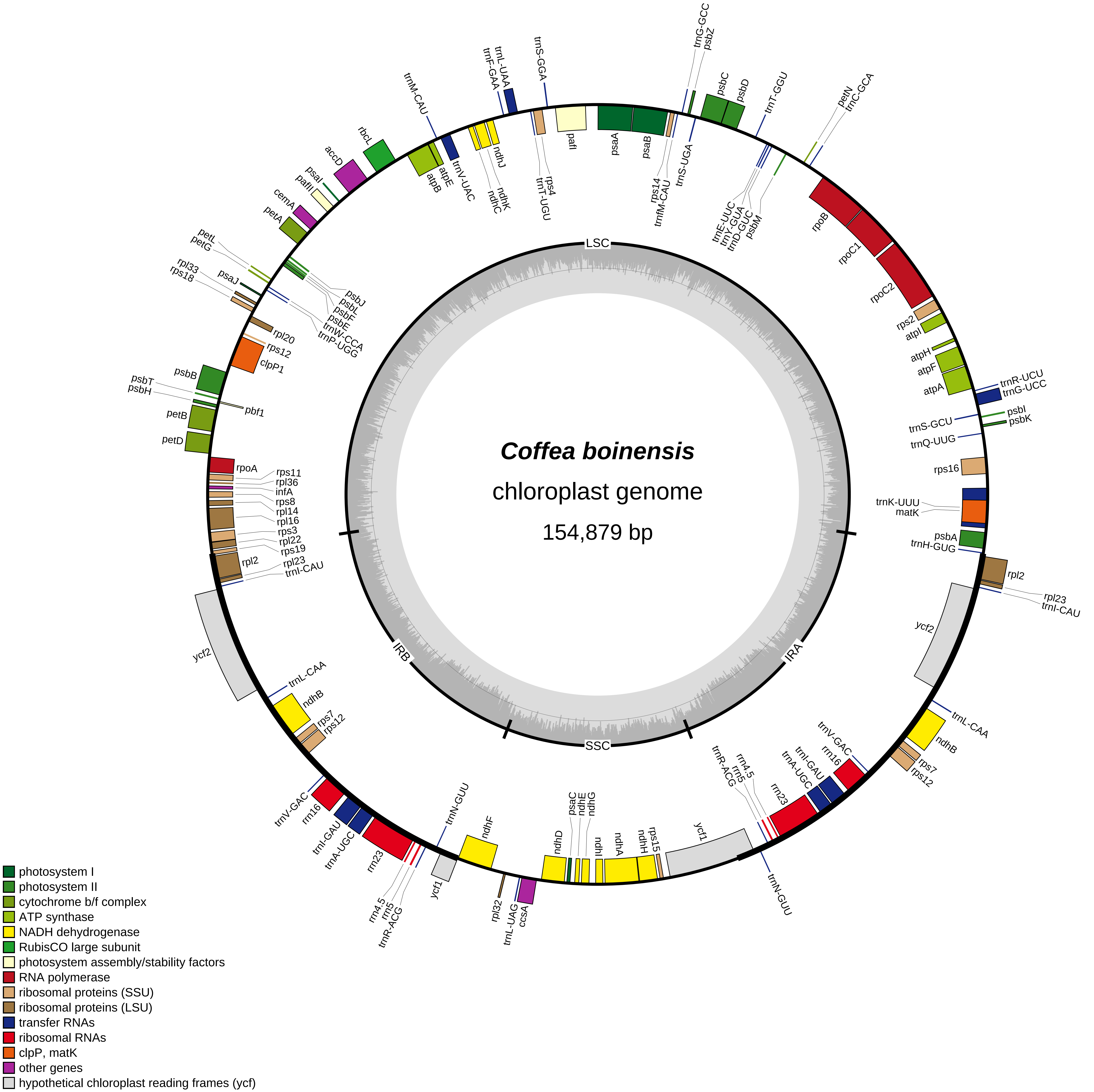

Coffea boinensis
chloroplast genome
154,879 bp
LSC
IRA
SSC
IRB
trnG-GCC
psbZ
psbC
psbD
trnT-GGU
petN
trnC-GCA
trnS-GGA
trnL-UAA
trnF-GAA
trnM-CAU
rbcL
accD
psaI
pafII
cemA
petA
petL
petG
rpl33
rps18
psaJ
rpl20
rps12
clpP1
psbB
psbT
psbH
pbf1
petB
petD
rpoA
rps11
rpl36
infA
rps8
rpl14
rpl16
rps3
rpl22
rps19
rpl2
rpl23
trnI-CAU
ycf2
trnL-CAA
ndhB
rps7
rps12
trnV-GAC
rrn16
trnI-GAU
trnA-UGC
rrn23
rrn4.5
rrn5
trnR-ACG
trnN-GUU
ycf1
ndhF
rpl32
trnL-UAG
ccsA
ndhD
psaC
ndhE
ndhG
ndhI
ndhA
ndhH
rps15
ycf1
trnN-GUU
trnR-ACG
rrn5
rrn4.5
rrn23
trnA-UGC
trnI-GAU
rrn16
trnV-GAC
rps12
rps7
ndhB
trnL-CAA
ycf2
rpl23
trnI-CAU
rpl2
trnH-GUG
psbA
matK
trnK-UUU
rps16
trnQ-UUG
trnS-GCU
psbI
psbK
trnR-UCU
trnG-UCC
atpA
atpF
atpH
atpI
rps2
rpoC2
rpoC1
rpoB
psbM
trnD-GUC
trnY-GUA
trnE-UUC
trnS-UGA
trnfM-CAU
rps14
psaB
psaA
pafI
rps4
trnT-UGU
ndhJ
ndhK
ndhC
trnV-UAC
atpE
atpB
psbJ
psbL
psbF
psbE
trnW-CCA
trnP-UGG
photosystem I
photosystem II
cytochrome b/f complex
ATP synthase
NADH dehydrogenase
RubisCO large subunit
photosystem assembly/stability factors
RNA polymerase
ribosomal proteins (SSU)
ribosomal proteins (LSU)
transfer RNAs
ribosomal RNAs
clpP, matK
other genes
hypothetical chloroplast reading frames (ycf)

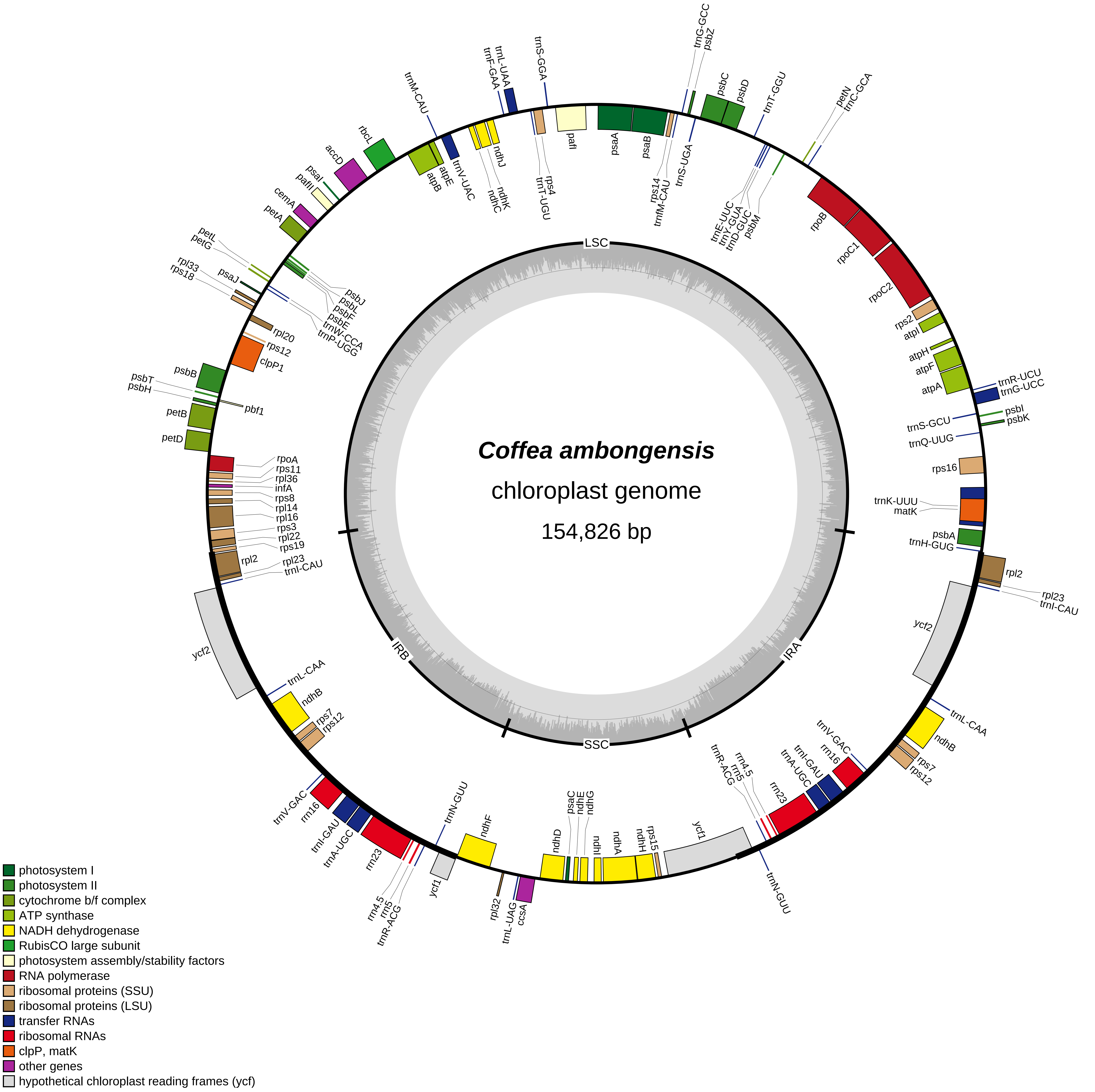

Coffea ambongensis
chloroplast genome
154,826 bp
LSC
IRA
SSC
IRB
trnM-CAU
trnL-UAA
trnF-GAA
trnS-GGA
trnG-GCC
psbZ
psbC
psbD
trnT-GGU
petN
trnC-GCA
rbcL
accD
psaI
pafII
cemA
petA
atpB
atpE
trnV-UAC
ndhC
ndhK
ndhJ
rps4
trnT-UGU
pafI
psaA
psaB
rps14
trnfM-CAU
trnS-UGA
trnE-UUC
trnY-GUA
trnD-GUC
psbM
rpoB
rpoC1
rpoC2
rps2
atpI
atpH
atpF
atpA
trnR-UCU
trnG-UCC
psbI
psbK
trnS-GCU
trnQ-UUG
rps16
trnK-UUU
matK
psbA
trnH-GUG
rpl2
rpl23
trnI-CAU
ycf2
trnL-CAA
ndhB
rps7
rps12
trnV-GAC
rrn16
trnI-GAU
trnA-UGC
rrn23
rrn4.5
rrn5
trnR-ACG
trnN-GUU
ycf1
rps15
ndhH
ndhA
ndhI
ndhG
ndhE
psaC
ndhD
ccsA
trnL-UAG
rpl32
ndhF
petL
petG
psbJ
psbL
psbF
psbE
trnW-CCA
trnP-UGG
rpl33
rps18
psaJ
rpl20
clpP1
psbB
psbT
psbH
pbf1
petB
petD
rpoA
rps11
rpl36
infA
rps8
rpl14
rpl16
rps3
rpl22
rps19
photosystem I
photosystem II
cytochrome b/f complex
ATP synthase
NADH dehydrogenase
RubisCO large subunit
photosystem assembly/stability factors
RNA polymerase
ribosomal proteins (SSU)
ribosomal proteins (LSU)
transfer RNAs
ribosomal RNAs
clpP, matK
other genes
hypothetical chloroplast reading frames (ycf)
