# Supplementary material for: The evolutionary history of three Baracoffea species from western Madagascar revealed by chloroplast and nuclear genomes
Source: PLoS One. 2024 Jan 11;19(1):e0296362. doi: 10.1371/journal.pone.0296362 (PMC10783717; doi:10.1371/journal.pone.0296362)
Supplement: S4 File — (PDF) [file pone.0296362.s004.pdf]

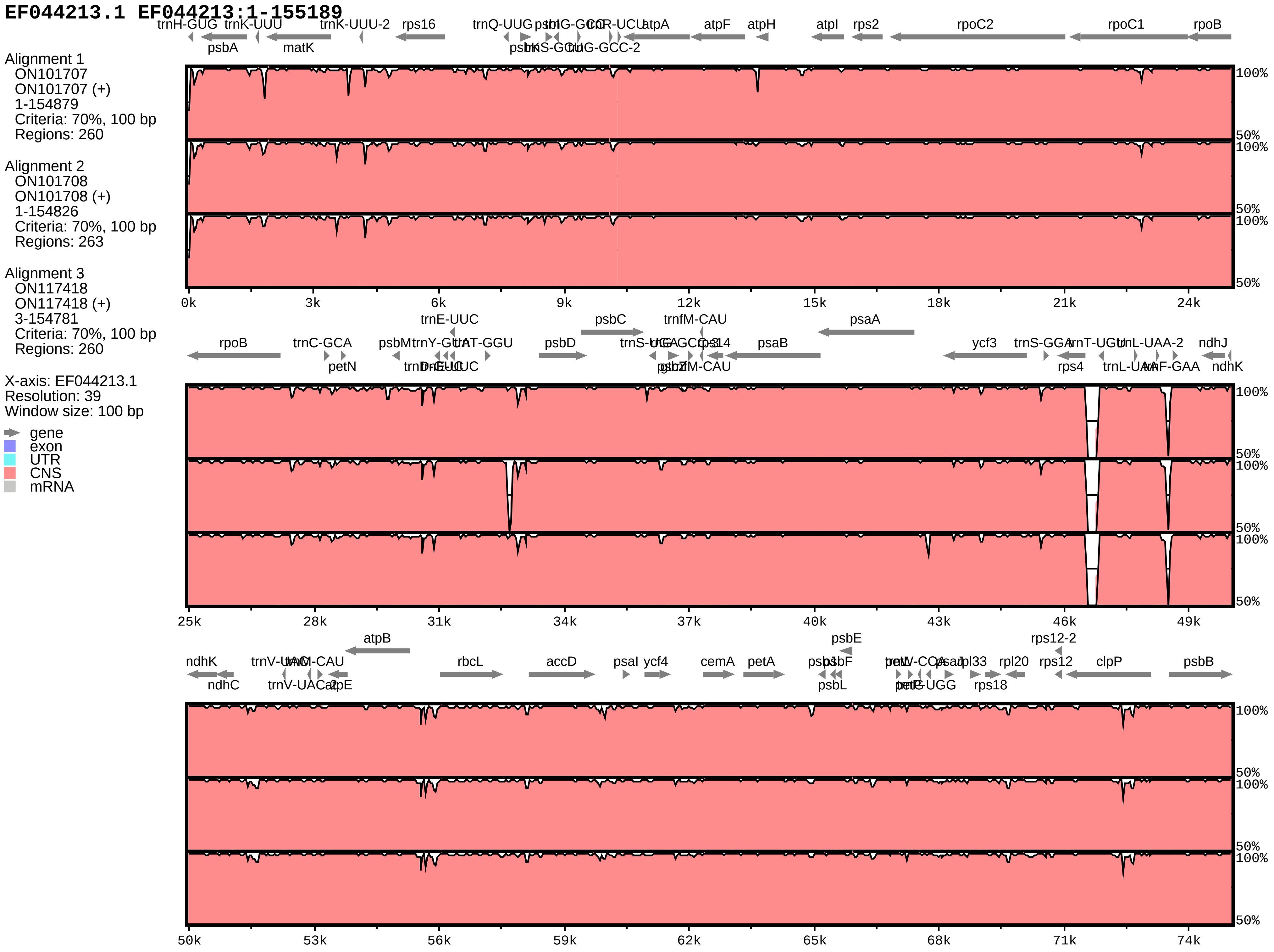
EF044213.1 EF044213:1-155189
Alignment 1
ON101707
ON101707 (+)
1-154879
Criteria: 70%, 100 bp
Regions: 260
Alignment 2
ON101708
ON101708 (+)
1-154826
Criteria: 70%, 100 bp
Regions: 263
Alignment 3
ON117418
ON117418 (+)
3-154781
Criteria: 70%, 100 bp
Regions: 260
X-axis: EF044213.1
Resolution: 39
Window size: 100 bp
gene
exon
UTR
CNS
mRNA
trnH-GUG
trnK-UUU
trnK-UUU-2
rps16
trnQ-UUG
atpA
atpF
atpH
atpI
rps2
rpoC2
rpoC1
rpoB
psbA
matK
100%
50%
0k
3k
6k
9k
12k
15k
18k
21k
24k
trnE-UUC
psbC
trnfM-CAU
psaA
trnC-GCA
psbD
psaB
ycf3
ndhJ
petN
rps4
ndhK
25k
28k
31k
34k
37k
40k
43k
46k
49k
atpB
psbE
rps12-2
ndhK
rbcL
accD
psaI
ycf4
cemA
petA
rpl20
rps12
clpP
psbB
ndhC
psbL
rps18
50k
53k
56k
59k
62k
65k
68k
71k
74k

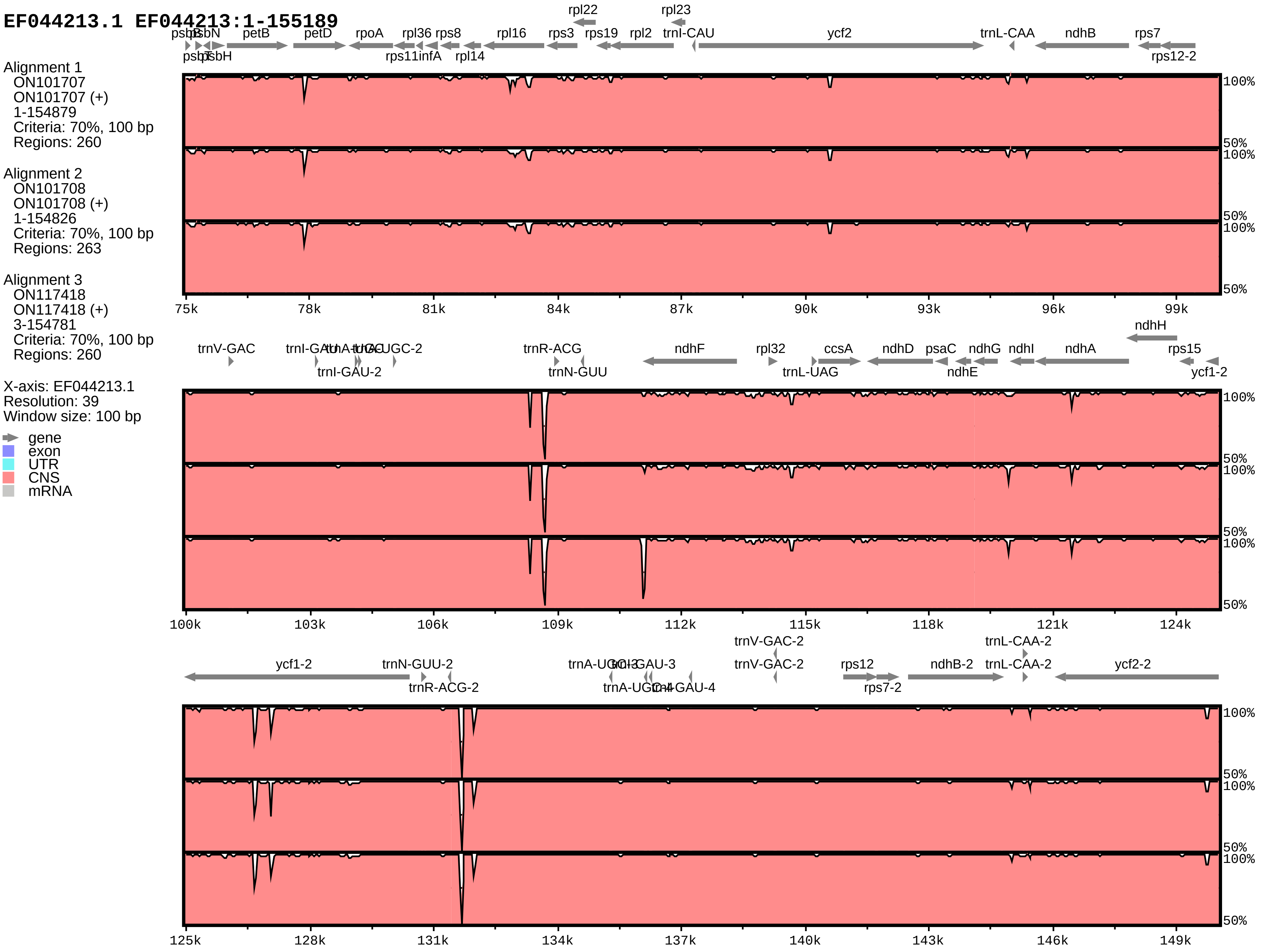
EF044213.1 EF044213:1-155189
Alignment 1
ON101707
ON101707 (+)
1-154879
Criteria: 70%, 100 bp
Regions: 260
Alignment 2
ON101708
ON101708 (+)
1-154826
Criteria: 70%, 100 bp
Regions: 263
Alignment 3
ON117418
ON117418 (+)
3-154781
Criteria: 70%, 100 bp
Regions: 260
X-axis: EF044213.1
Resolution: 39
Window size: 100 bp
gene
exon
UTR
CNS
mRNA
rpl22
rpl23
petB
petD
rpoA
rpl36
rps8
rpl16
rps3
rps19
rpl2
trnI-CAU
ycf2
trnL-CAA
ndhB
rps7
psbH
rps11
infA
rpl14
rps12-2
100%
50%
75k
78k
81k
84k
87k
90k
93k
96k
99k
ndhH
trnV-GAC
trnR-ACG
ndhF
rpl32
ccsA
ndhD
psaC
ndhG
ndhI
ndhA
rps15
trnI-GAU-2
trnN-GUU
trnL-UAG
ndhE
ycf1-2
100k
103k
106k
109k
112k
115k
118k
121k
124k
trnV-GAC-2
trnL-CAA-2
ycf1-2
trnN-GUU-2
rps12
ndhB-2
ycf2-2
trnR-ACG-2
rps7-2
125k
128k
131k
134k
137k
140k
143k
146k
149k

EF044213.1 EF044213:1-155189

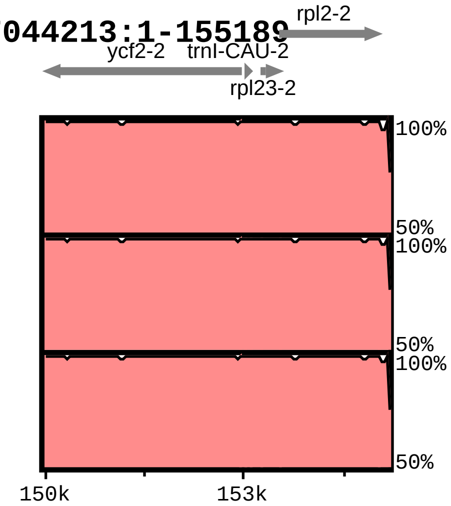

Alignment 1
ON101707
ON101707 (+)
1-154879
Criteria: 70%, 100 bp
Regions: 260

Alignment 2
ON101708
ON101708 (+)
1-154826
Criteria: 70%, 100 bp
Regions: 263

Alignment 3
ON117418
ON117418 (+)
3-154781
Criteria: 70%, 100 bp
Regions: 260

X-axis: EF044213.1
Resolution: 39
Window size: 100 bp

gene
exon
UTR
CNS
mRNA
