# Supplementary figures and images for: The evolutionary history of three Baracoffea species from western Madagascar revealed by chloroplast and nuclear genomes
Source: PLoS One. 2024 Jan 11;19(1):e0296362. doi: 10.1371/journal.pone.0296362 (PMC10783717; doi:10.1371/journal.pone.0296362)

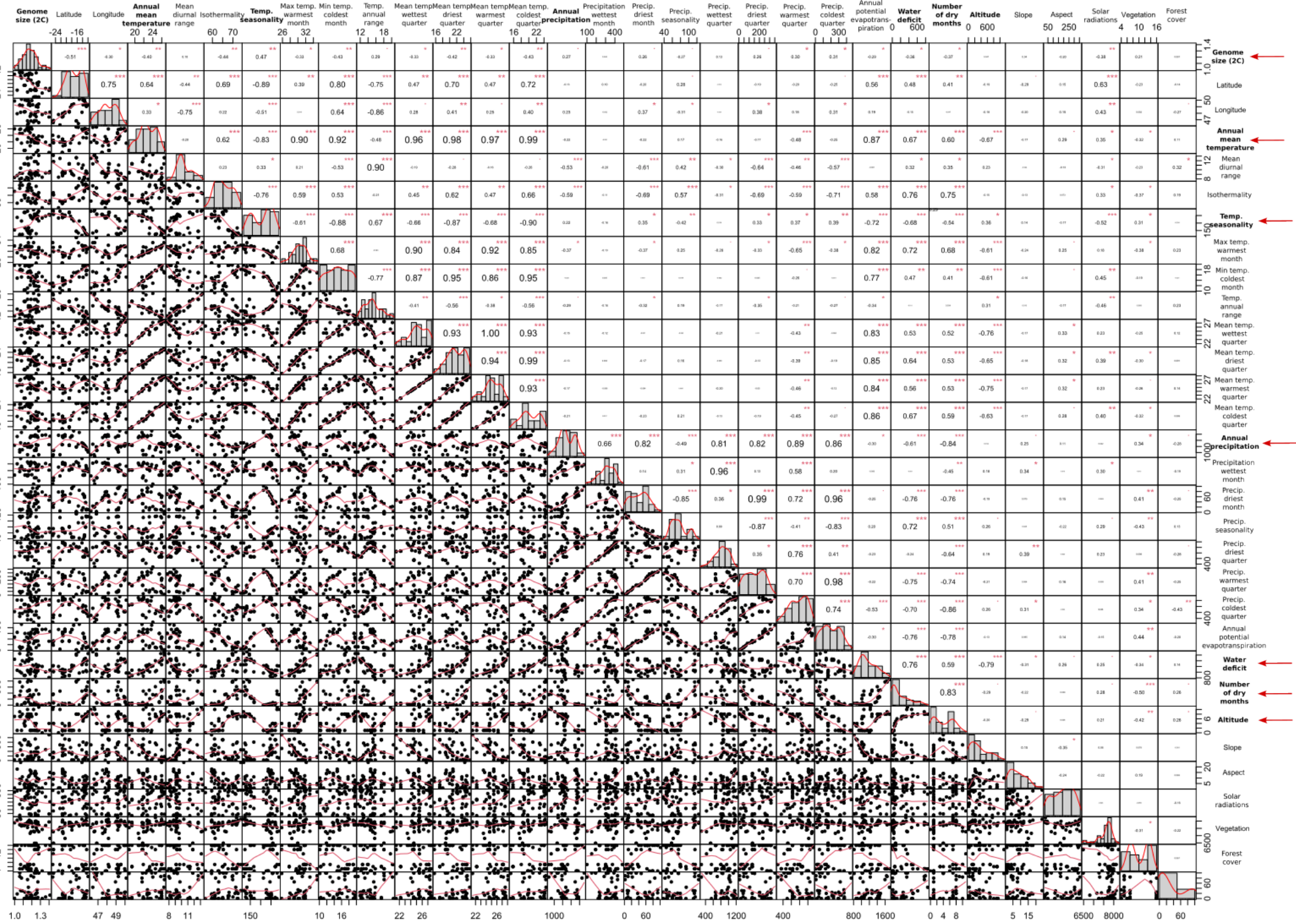

Supplement: S6 File — (PDF) [file pone.0296362.s006.pdf]

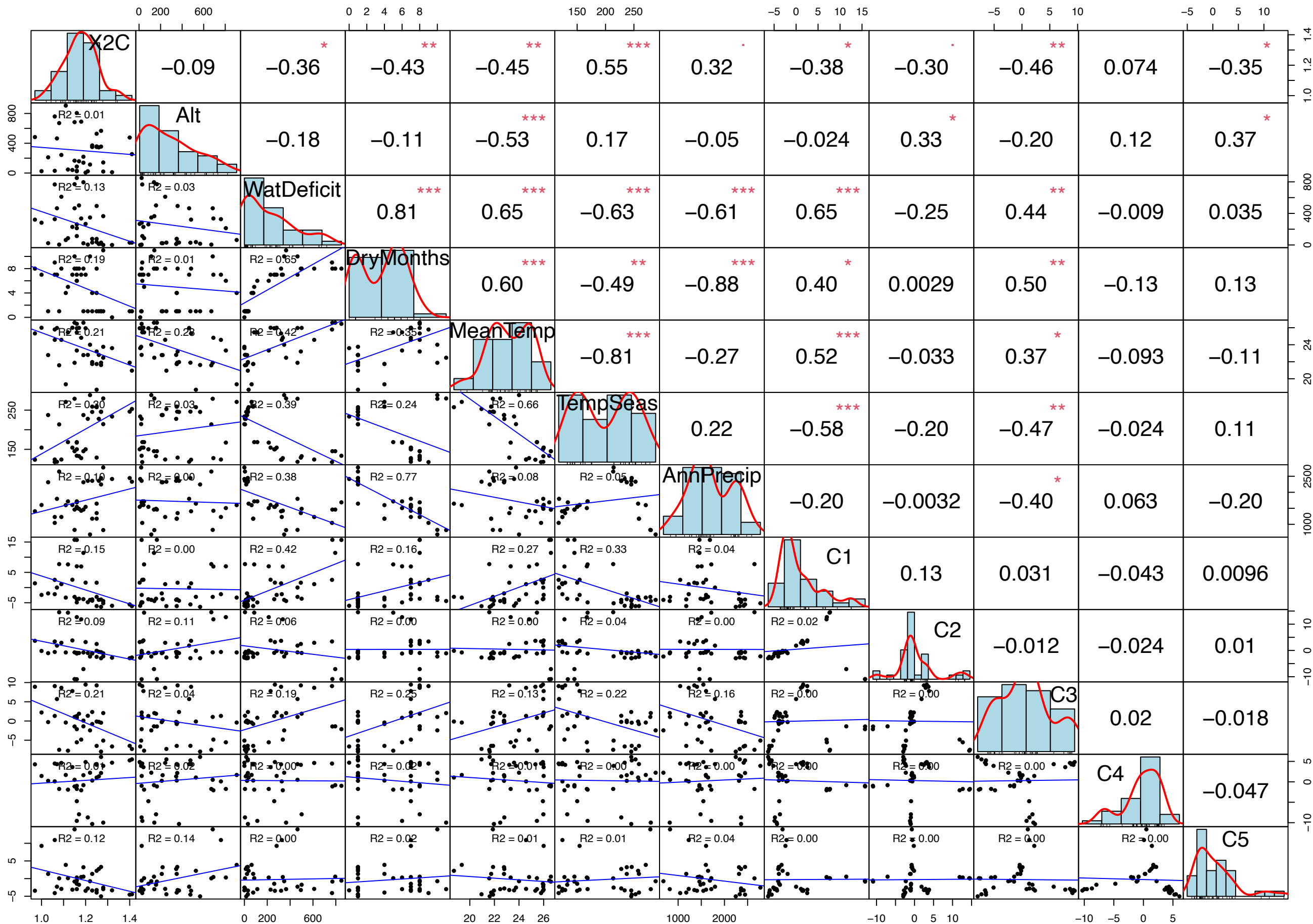

Supplement: S8 File — (PDF) [file pone.0296362.s008.pdf]
