# Supplementary material for: The evolutionary history of three Baracoffea species from western Madagascar revealed by chloroplast and nuclear genomes
Source: PLoS One. 2024 Jan 11;19(1):e0296362. doi: 10.1371/journal.pone.0296362 (PMC10783717; doi:10.1371/journal.pone.0296362)

Genome size ~ Latitude

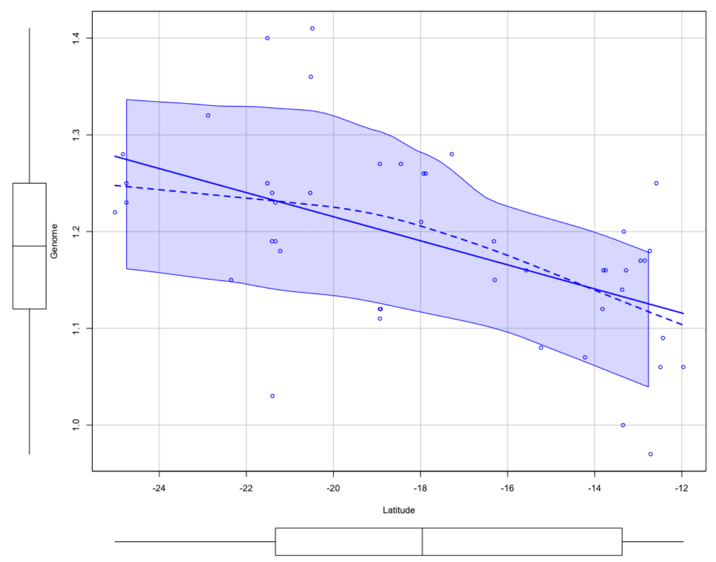

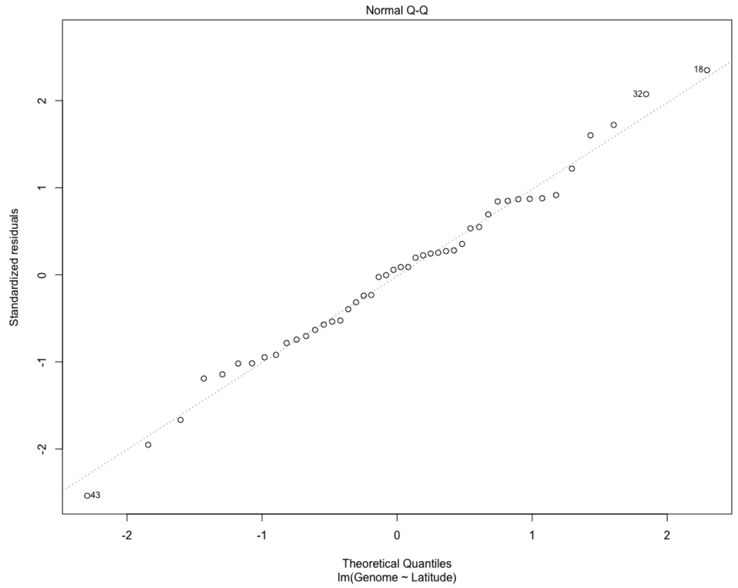

Shapiro-Wilk normality test

data: residuals(GL.lm1)
W = 0.99014, p-value = 0.9616

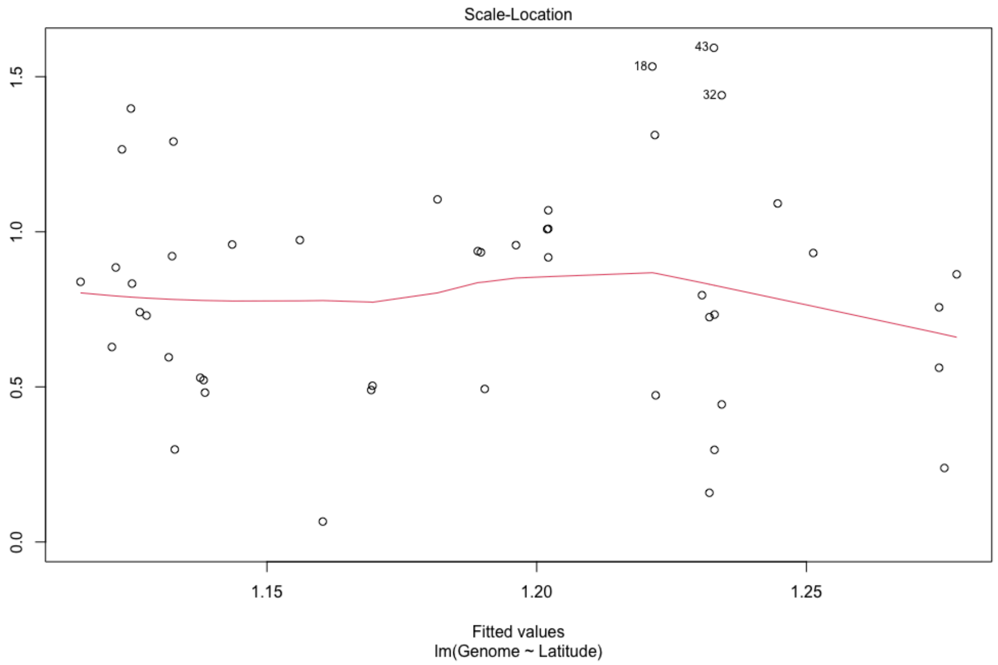

Non constant Variance score test

Variance formula: ~ fitted.values
Chisquare = 0.3821315, Df =1, p= 0.53646

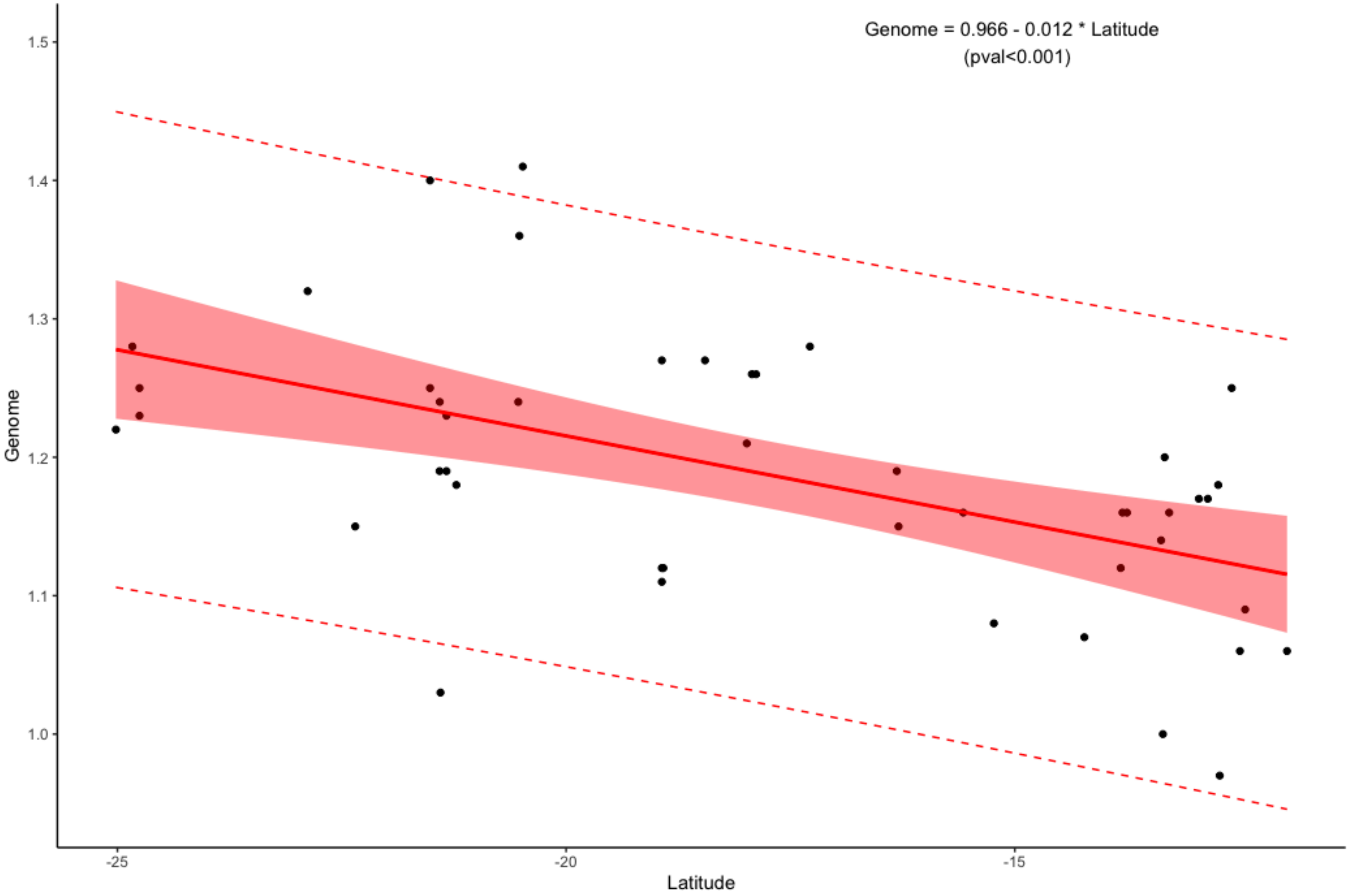

Genome size ~Number of Dry months

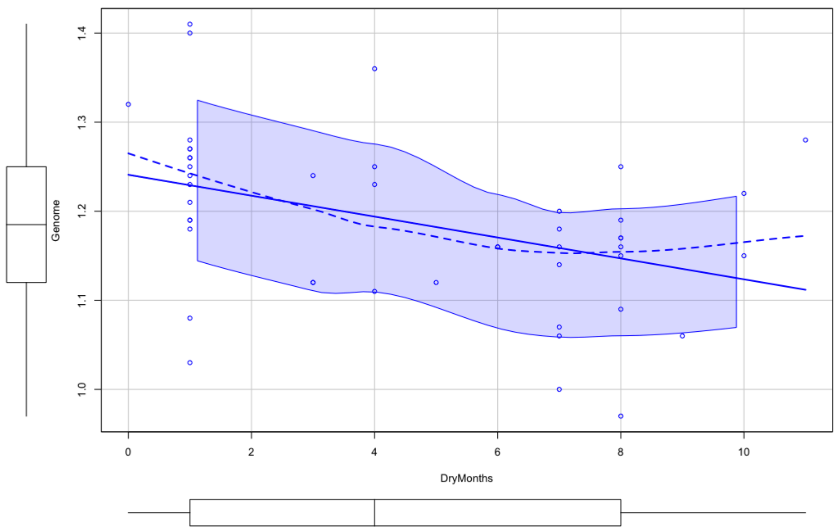

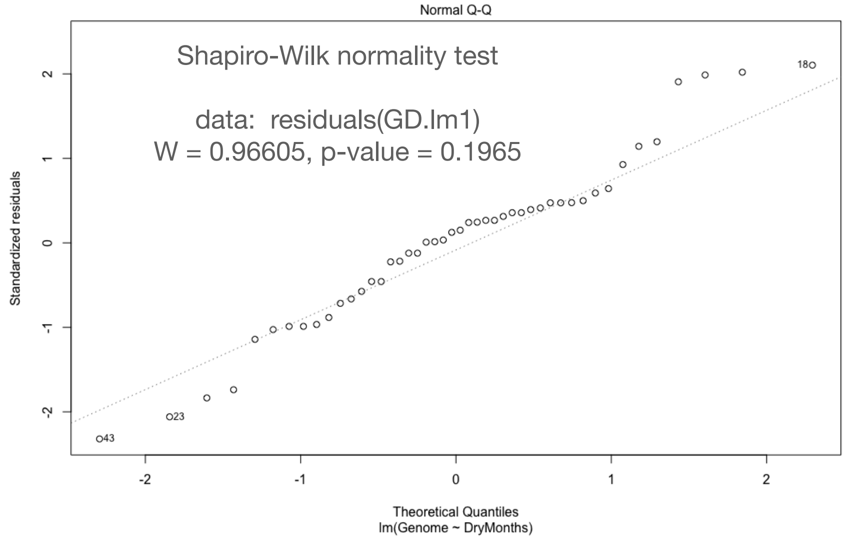

Shapiro-Wilk normality test

data: residuals(GD.lm1)
W = 0.96605, p-value = 0.1965

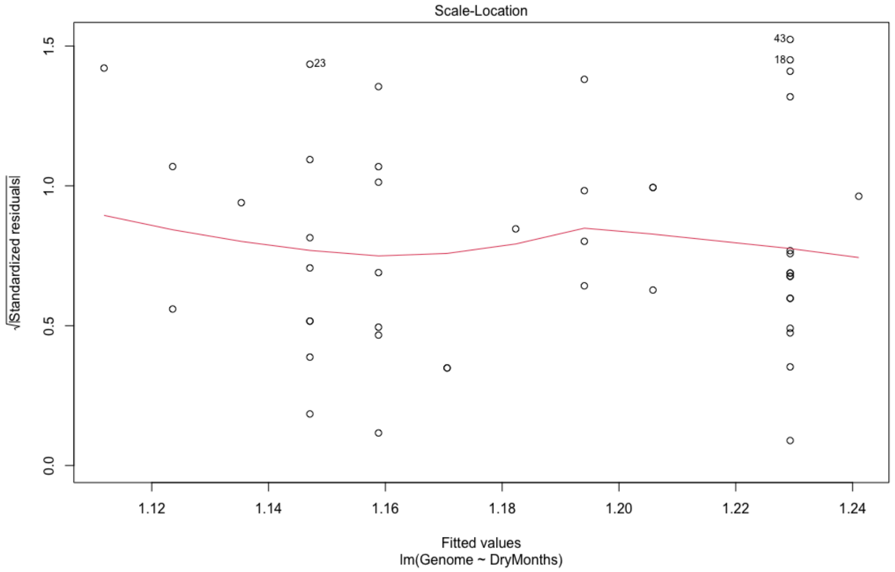

Non constant Variance score test

Variance formula: ~ fitted.values
Chisquare = 0.0226488, Df =1, p= 0.88037

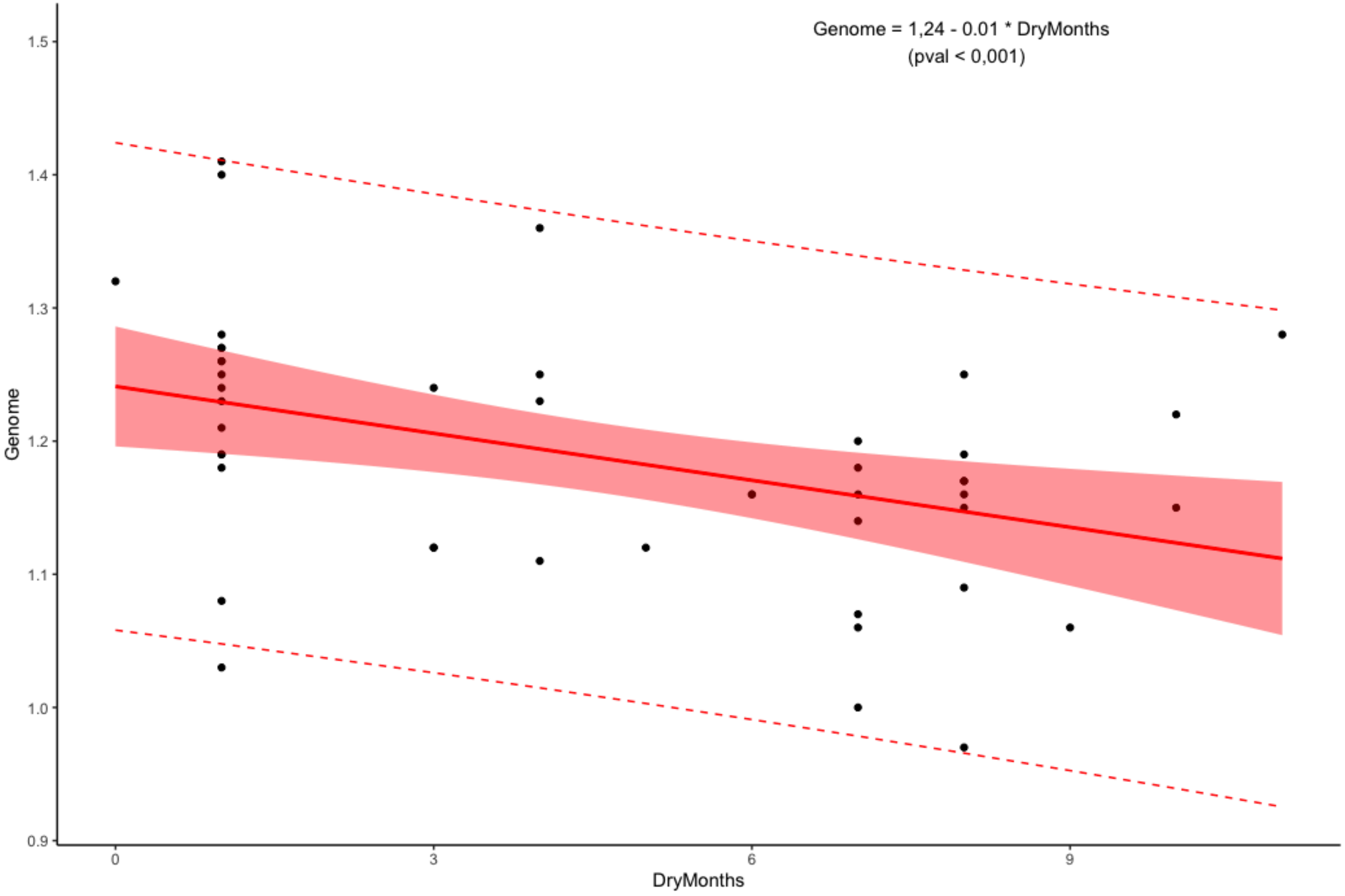

Genome = 1,24 - 0.01 * DryMonths
(pval < 0,001)

Genome size ~Temperature Seasonability

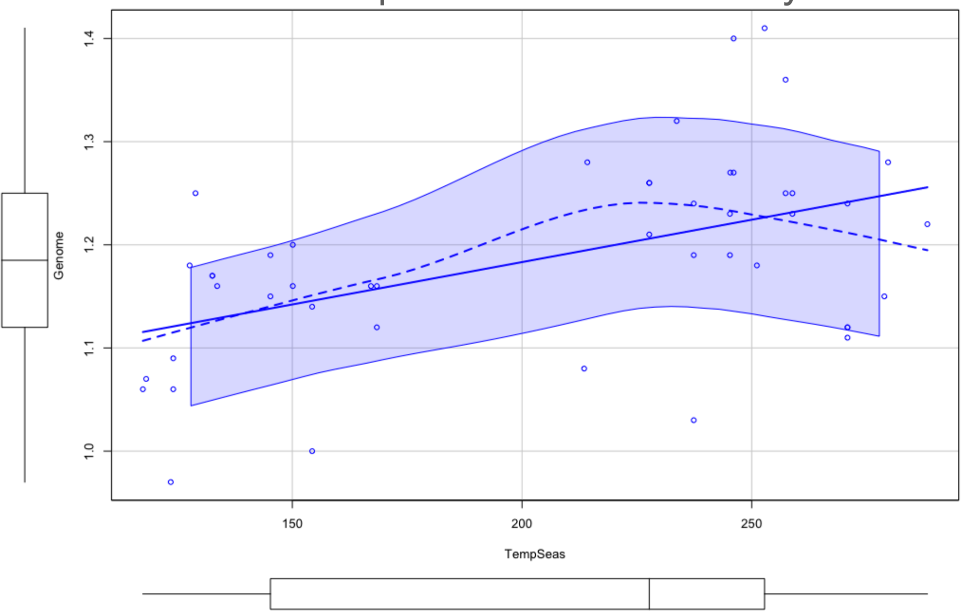

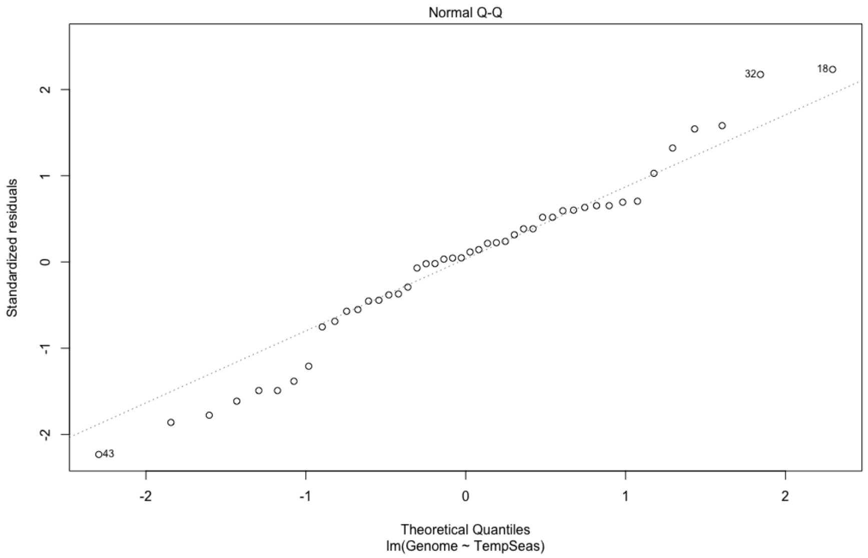

Shapiro-Wilk normality test

data: residuals(GTS.lm1)
W = 0.97278, p-value = 0.35

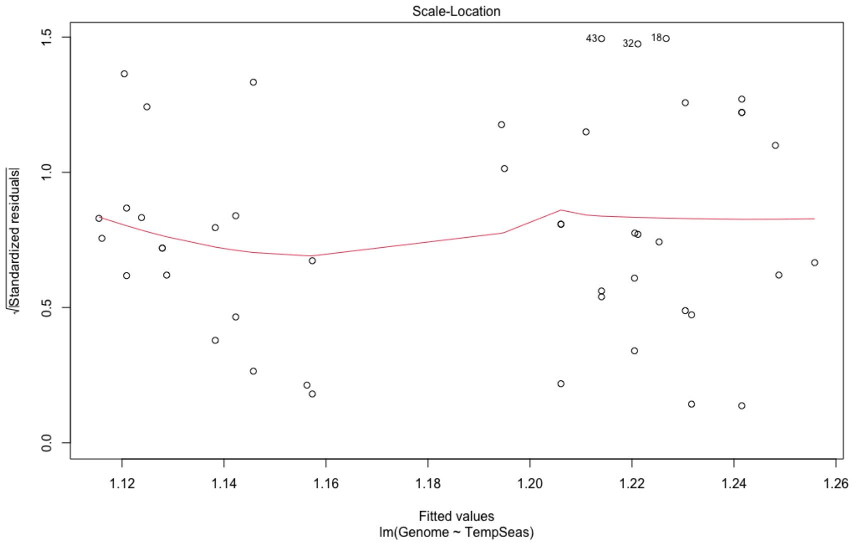

Non constant Variance score test

Variance formula: ~ fitted.values
Chisquare = 1.245027, Df = 1, p = 0.2645

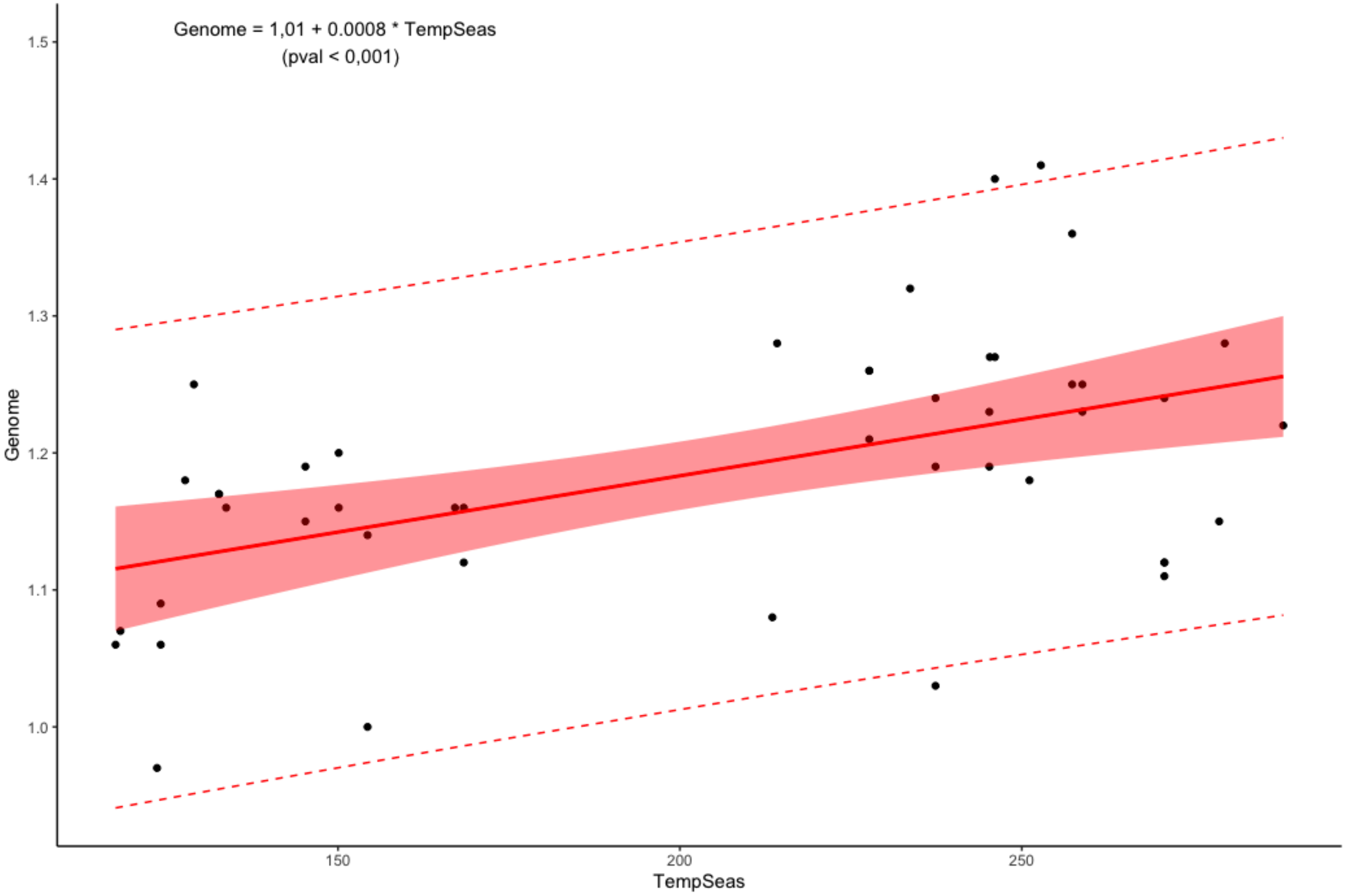

Genome size ~ Water Deficit

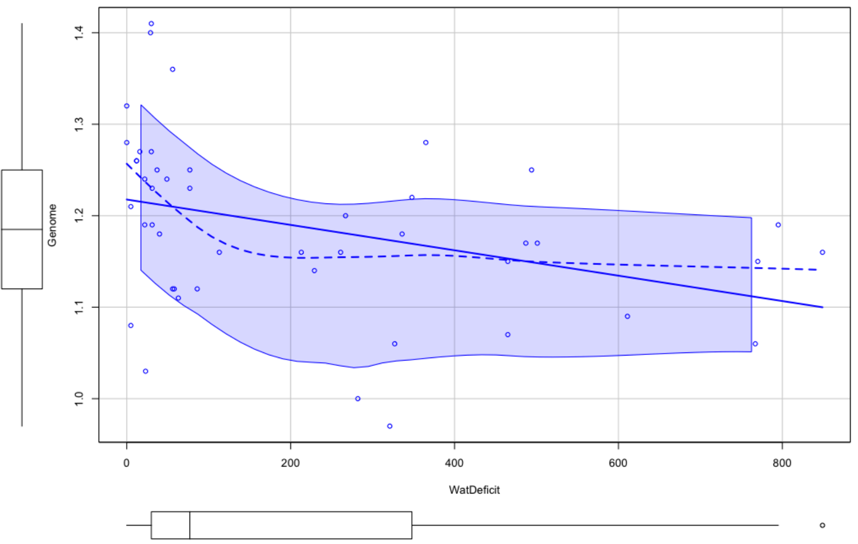

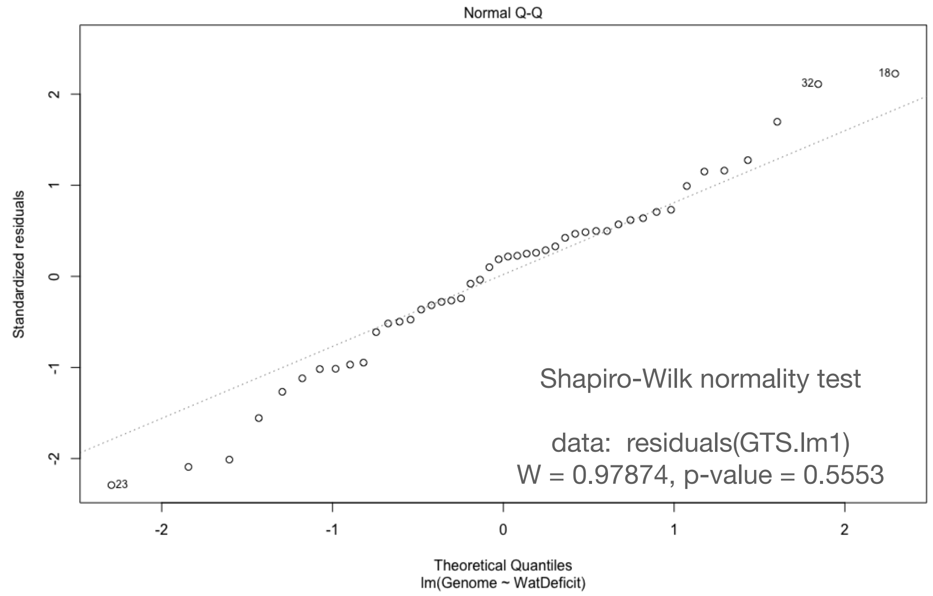

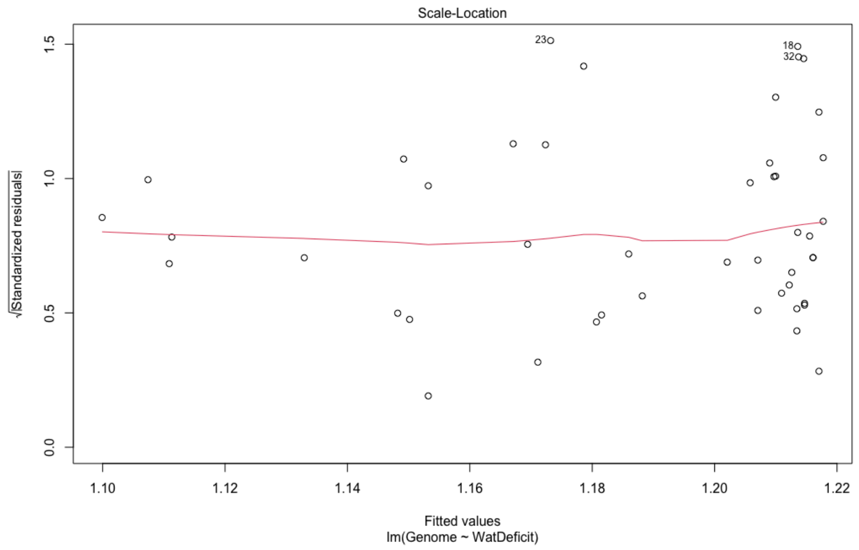

Non constant Variance score test

Variance formula: ~ fitted.values
Chisquare = 0.8377181, Df = 1,
p = 0.36005

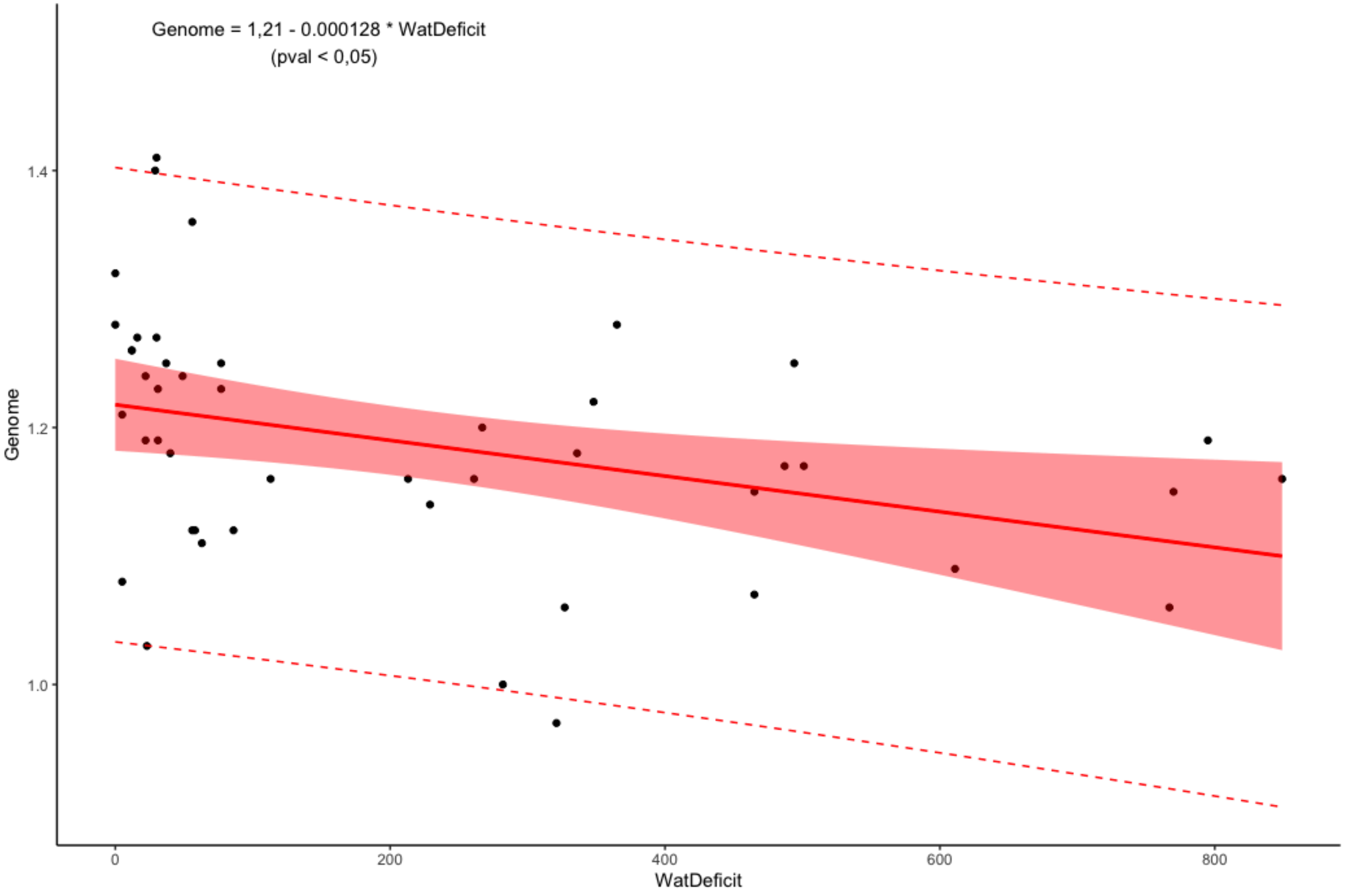

Genome size ~ Annual Precipitation

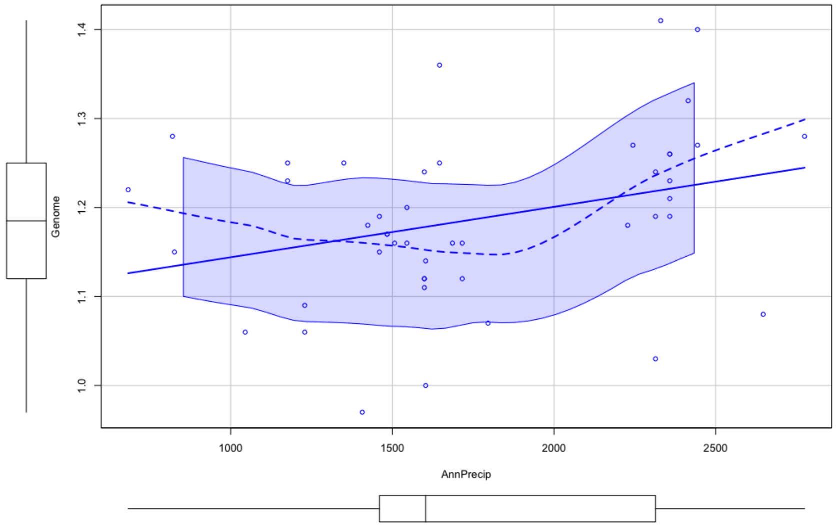

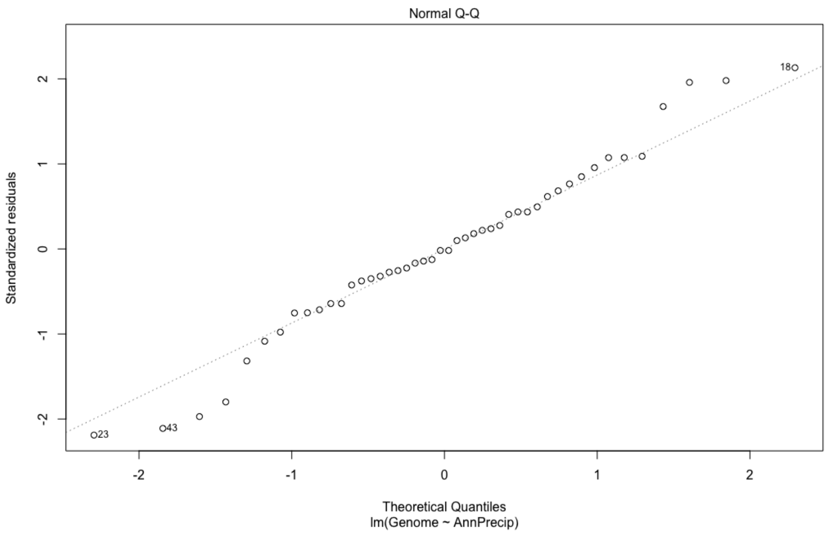

Shapiro-Wilk normality test

data: residuals(GTS.lm1)
W = 0.97801, p-value = 0.527

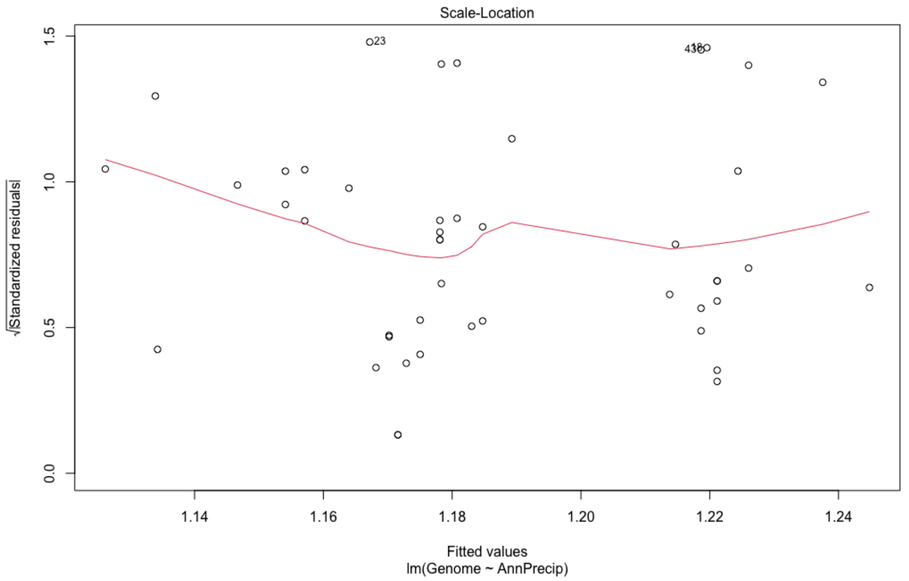

Non constant Variance score test

Variance formula: ~ fitted.values
Chisquare = 0.1821223, Df = 1,
p = 0.66956

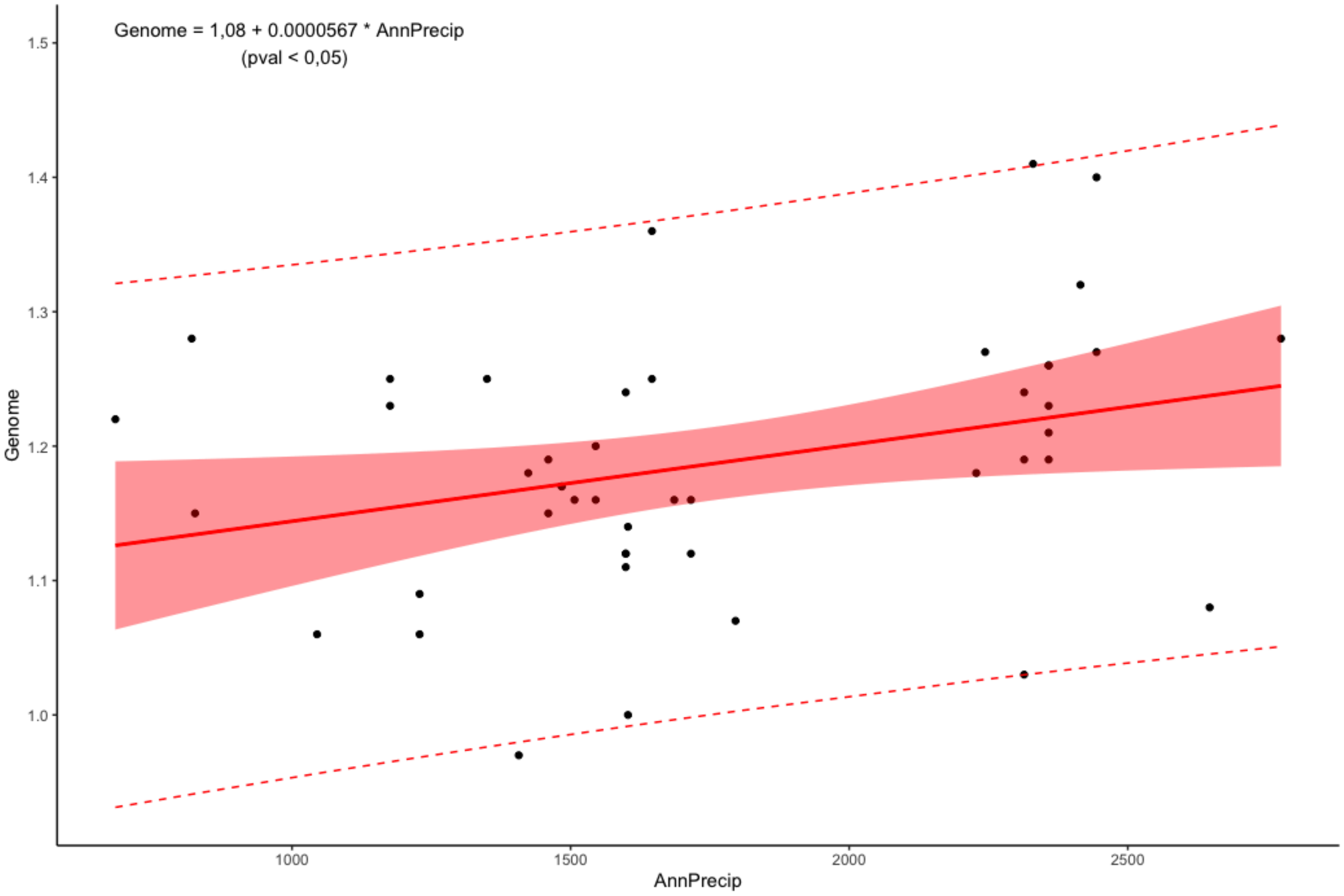

Genome size ~ Alt

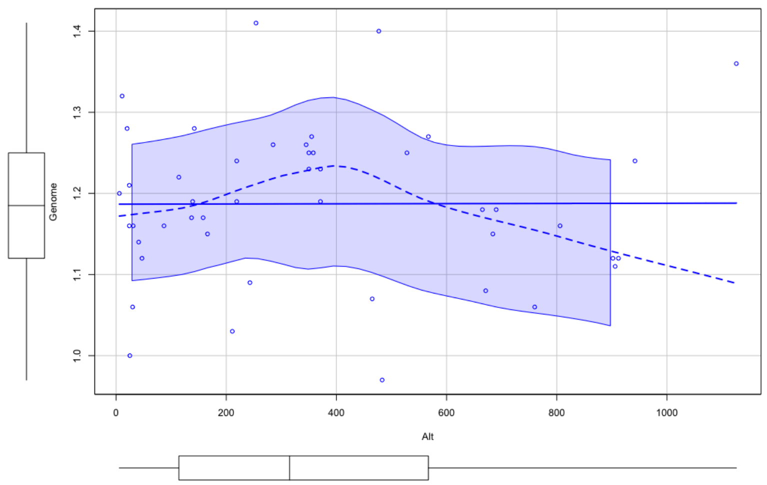

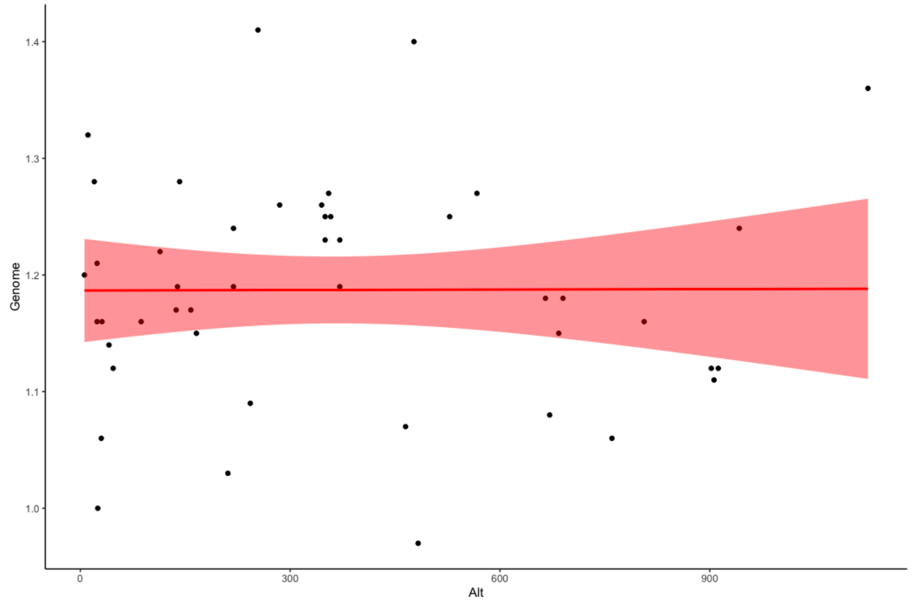

Genome size ~ Longitude

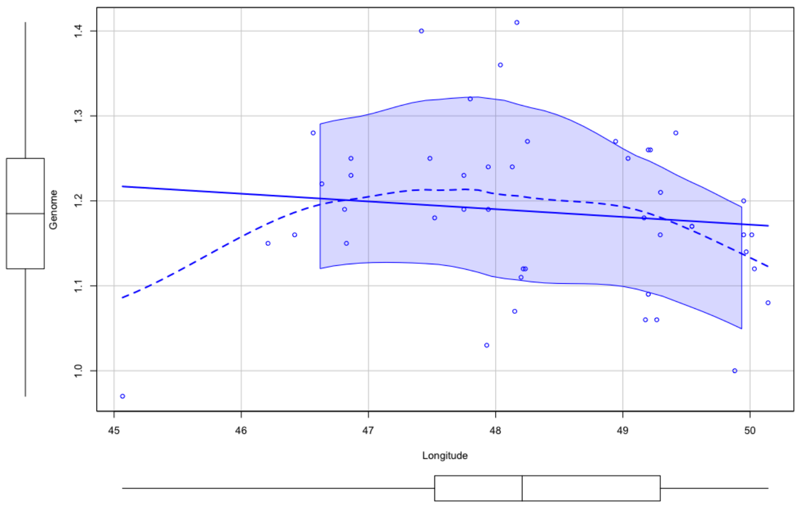

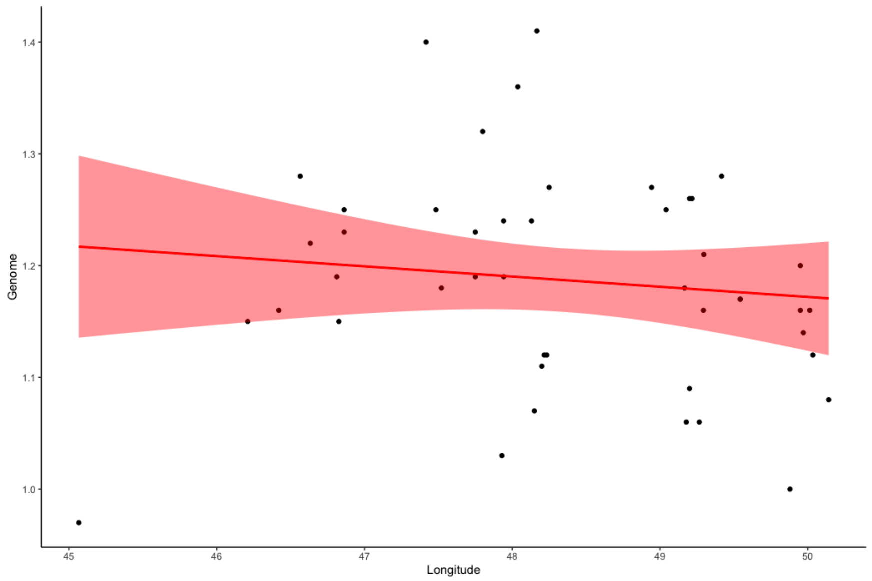

Genome size ~ Mean temperature

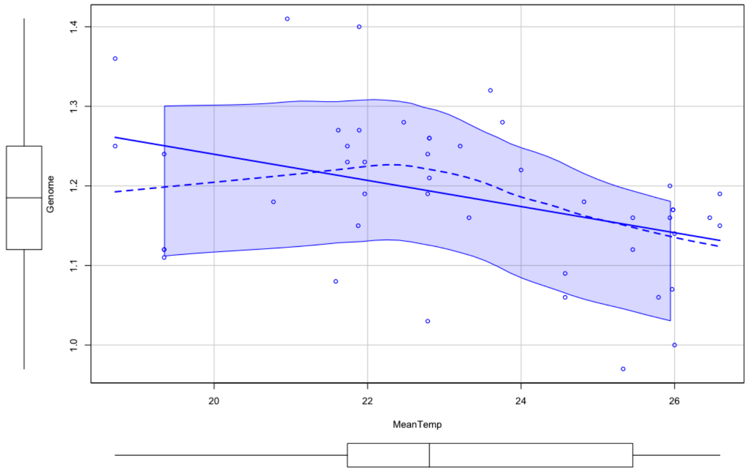

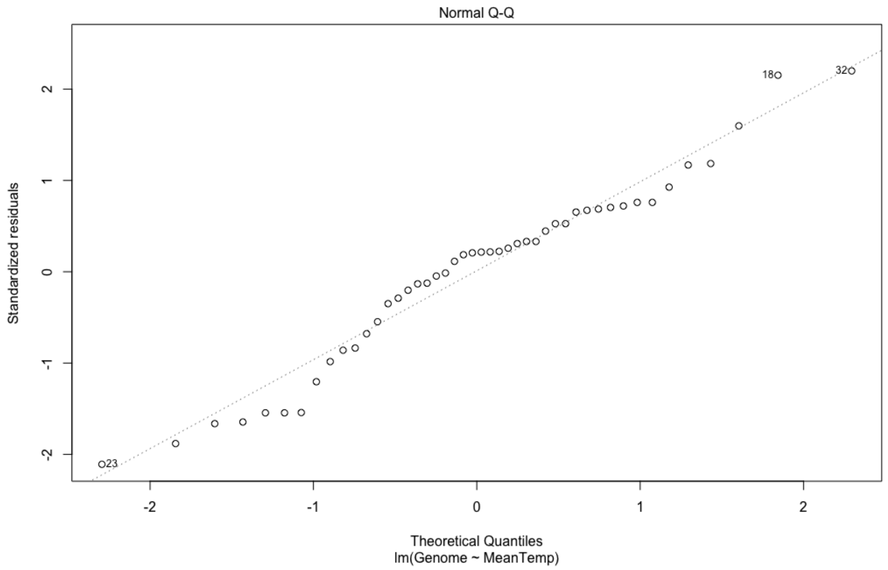

Shapiro-Wilk normality test

data: residuals(GTS.lm1)
W = 0.96495, p-value = 0.1782

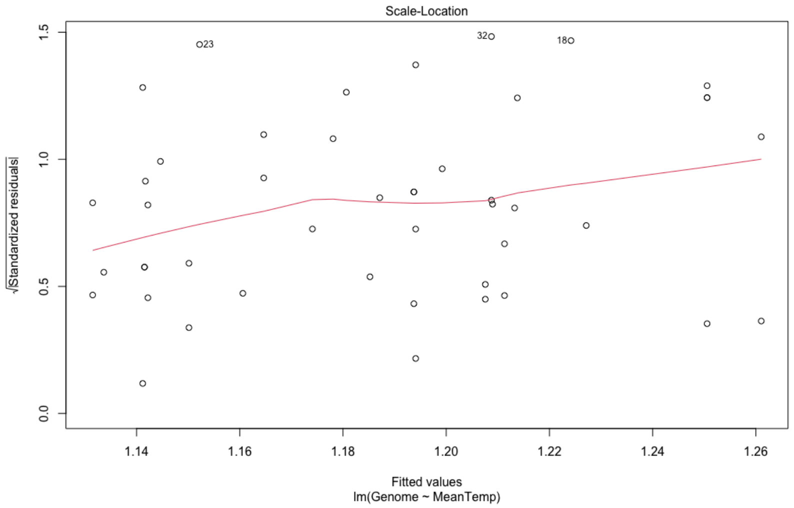

Non constant Variance score test

Variance formula: ~ fitted.values
Chisquare = 1.810929, Df = 1, p = 0.1784

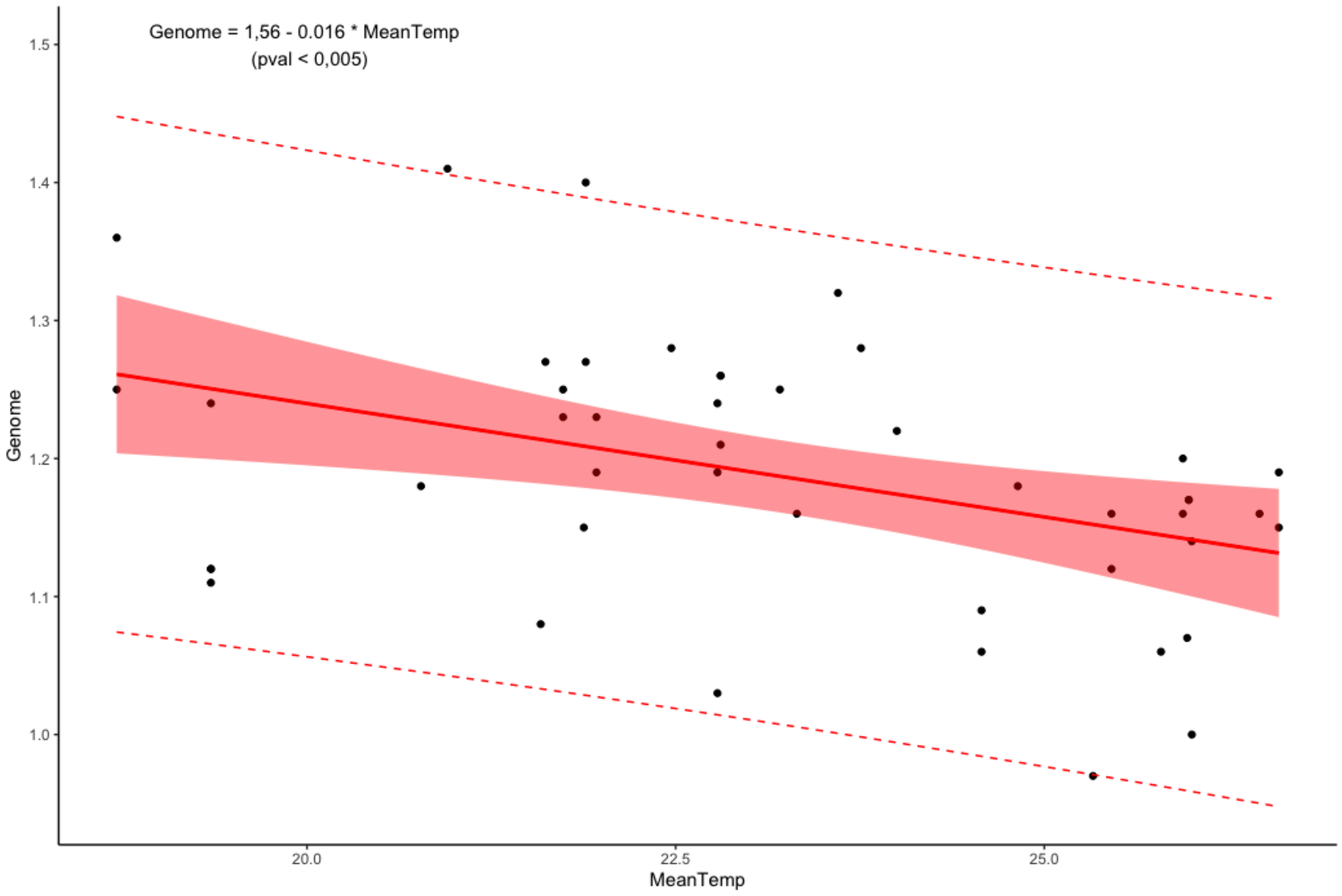

Genome size ~ Evap

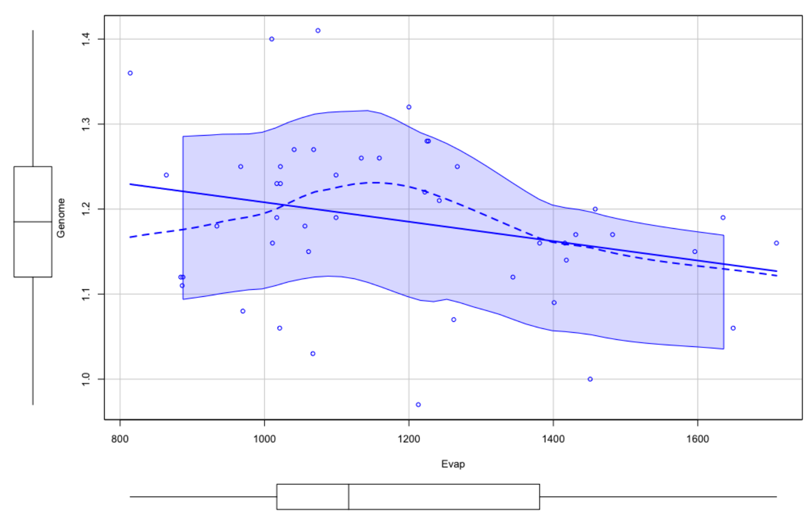

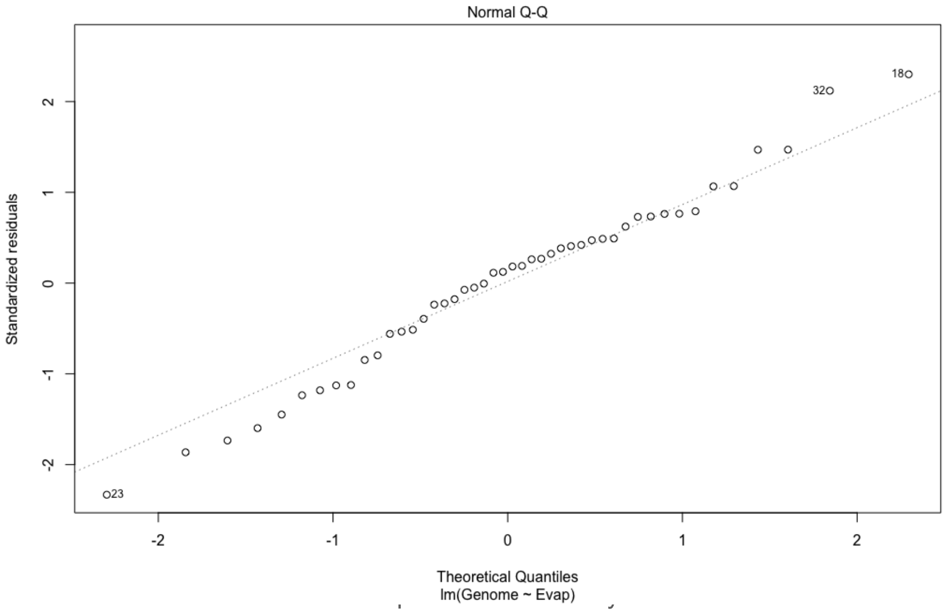

data: residuals(GTS.lm1)
W = 0.98299, p-value = 0.7297

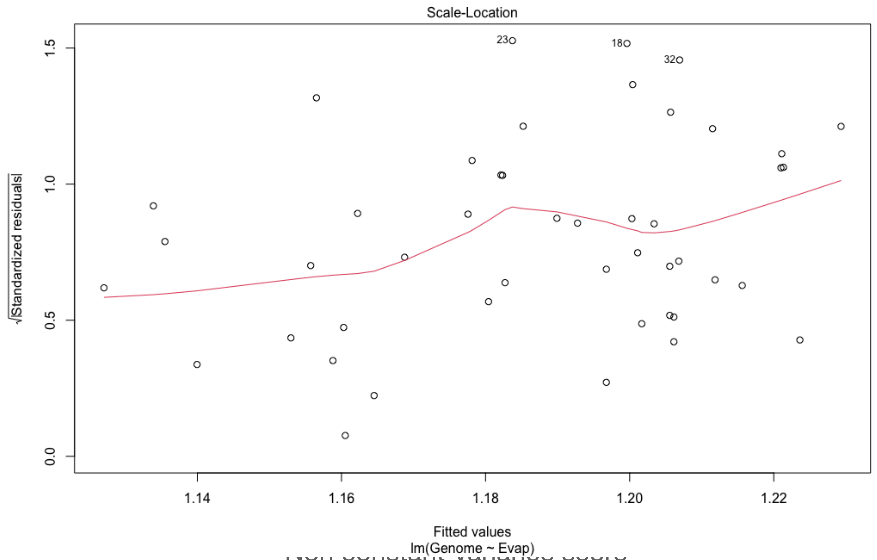

Non-constant variance score test

Variance formula: ~ fitted.values
Chisquare = 1.826612, Df = 1, p = 0.17653

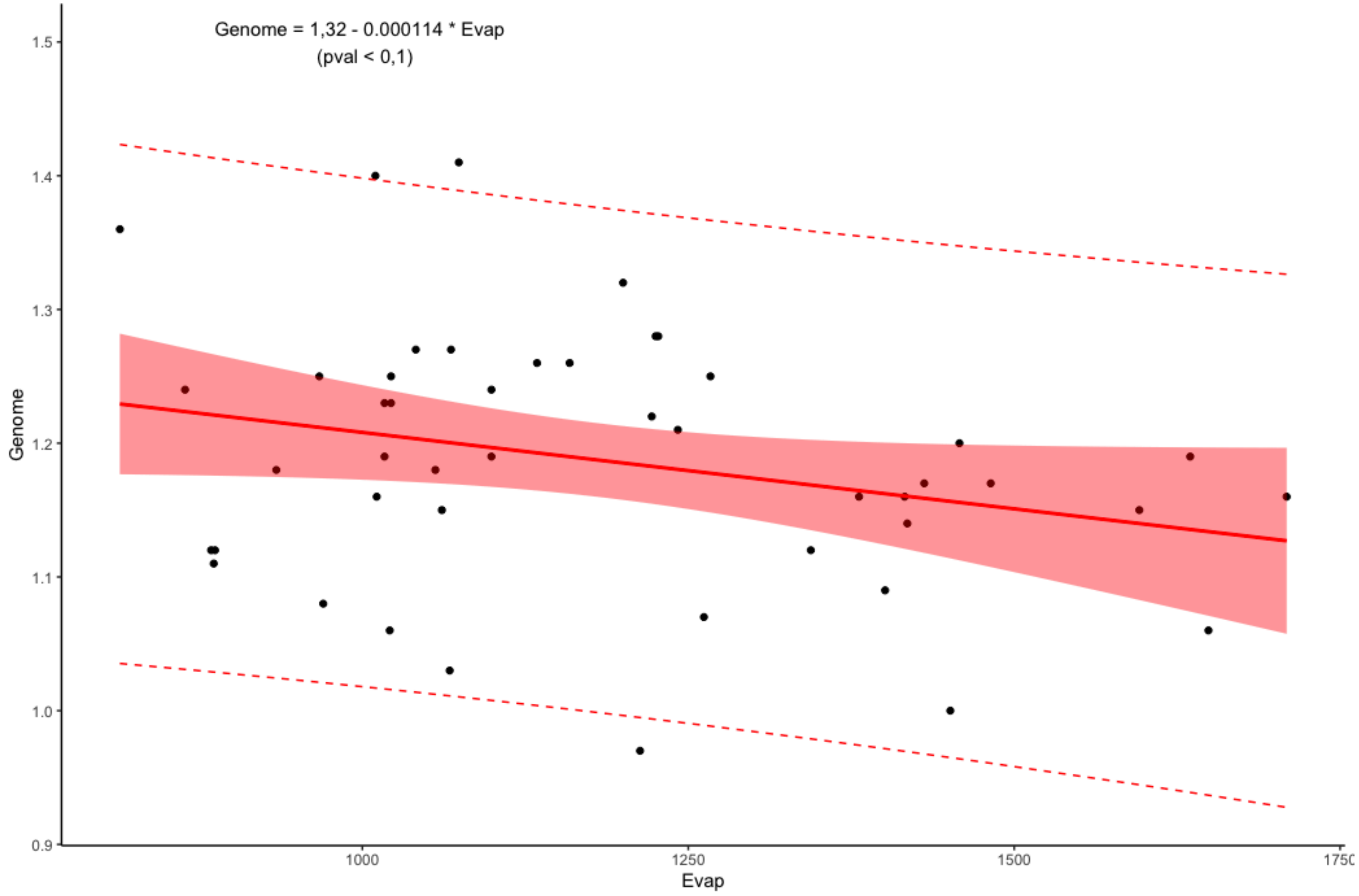

Supplement: S7 File — (PDF) [file pone.0296362.s007.pdf]
